# Assessing human exposure to pesticides and mycotoxins: optimization and validation of a method for multianalyte determination in urine samples

**DOI:** 10.1007/s00216-024-05191-2

**Published:** 2024-02-07

**Authors:** Jesús Marín-Sáez, Maykel Hernández-Mesa, Jose A. Gallardo-Ramos, Laura Gámiz-Gracia, Ana M. García-Campaña

**Affiliations:** 1 Department of Analytical Chemistry, Faculty of Sciences, University of Granada, Campus Fuentenueva S/N, 18071 Granada, Spain; 2 Research Group “Analytical Chemistry of Contaminants”, Department of Chemistry and Physics, Research Centre for Mediterranean Intensive Agrosystems and Agri-Food Biotechnology (CIAIMBITAL), University of Almeria, Agrifood Campus of International Excellence, ceiA3, 04120 Almeria, Spain; 3 Department of Food Technology, Engineering and Science, Applied Mycology Group, AGROTECNIO-CERCA Center, University of Lleida, 25198 Lleida, Spain

**Keywords:** Urine, Biomonitoring, Pesticides, Mycotoxins, SALLE, UHPLC-MS/MS

## Abstract

Humans are exposed to an increasing number of contaminants, with diet being one of the most important exposure routes. In this framework, human biomonitoring is considered the gold standard for evaluating human exposure to chemicals. Pesticides and mycotoxins are chemicals of special concern due to their health implications. They constitute the predominant border rejection notifications for food and feed in Europe and the USA. However, current biomonitoring studies are focused on a limited number of compounds and do not evaluate mycotoxins and pesticides together. In this study, an analytical method has been developed for the determination of 30 pesticides and 23 mycotoxins of concern in urine samples. A salting-out liquid–liquid extraction (SALLE) procedure was optimized achieving recoveries between 70 and 120% for almost all the compounds and limits as lower as when QuEChERS was applied. The compounds were then determined by liquid chromatography coupled to triple quadrupole mass spectrometry. Different chromatographic conditions and analytical columns were tested, selecting a Hypersild gold aQ column as the best option. Finally, the method was applied to the analysis of 45 urine samples, in which organophosphate and pyrethroid pesticides (detection rates (DR) of 82% and 42%, respectively) and ochratoxin A and deoxynivalenol (DR of 51% and 33%, respectively) were the most detected compounds. The proposed analytical method involves the simultaneous determination of a diverse set of pesticides and mycotoxins, including their most relevant metabolites, in human urine. It serves as an essential tool for biomonitoring the presence of highly prevalent contaminants in modern society.

**Graphical Abstract:**

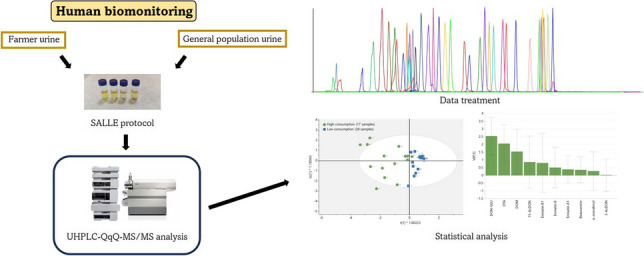

**Supplementary Information:**

The online version contains supplementary material available at 10.1007/s00216-024-05191-2.

## Introduction

Humans are subjected to an increasing number of exposures throughout their lives, including all the chemicals and compounds mainly coming from agricultural and industrial practices. In this sense, environmental exposures constitute a significant health risk, as they can cause severe health effects [1, 2]. About 350,000 compounds and mixtures are currently registered in chemical inventories, and about 69,000 chemical compounds are in commerce [3]. In addition, there are several natural toxins produced by living organisms that are not harmful to the organisms themselves, but they may be toxic to humans or animals, when eaten. This is the case of mycotoxins, produced by certain types of moulds that colonize crops, being dangerous to health [4]. Therefore, strict control of these substances is needed in food and environmental samples to ensure the health safety. Traditionally, this has been done through food and feed monitoring programs, such as those done by the Federal Drug Administration (FDA), in the USA, or the European Food Safety Authority (EFSA), in the European Union. This is so because the diet is considered the major exposure route to contaminants such as pesticides and mycotoxins [5–7].

However, although these controls are necessary to ensure food safety, they do not provide exposure information. Thus, some aspects such as individual exposure information, bioaccessible and bioavailability information, depending on the contaminants, as well as the individual differences in absorption, distribution, metabolism and excretion (ADME) of contaminants when an exposure is produced, are not available with this approach [8, 9]. In this matter, it is of utmost importance to establish human exposure to chemicals in order to study the effects of co-exposure of these compounds on human health, although it is currently under-researched [10]. For these reasons, chemical biomonitoring is an affordable alternative that aims to generate reliable exposure data by determining chemicals or their metabolites in biological specimens [11].

Biomonitoring studies are increasingly performed in combination with non-targeted metabolomics studies in an effort to link exposure and health outcomes [12]. However, there is a high risk of loss of exposure data when following this approach, as contaminants are typically found at very low concentrations compared to endogenous compounds (1000 times lower in some cases) [13]. Therefore, targeted methods are needed to truly characterize chemical exposure. Common biomonitoring programs focus on a few congeners of contaminants from the same or related families [14]. However, since humans are exposed to complex chemical mixtures, it is necessary to conduct a broader study evaluating compounds from different families simultaneously.

Among all the exposures that affect humans, pesticides are of great concern. The increasing use of them not only imply an improvement in food production, but also a concern in relation to negative environmental and health implications. Among the different classes of pesticides, some of the most employed worldwide are organophosphates (OPs), pyrethroids (PYs), neonicotinoids (NEOs) and some fungicides. They can cause serious health problems such as neurotoxicity, immunotoxicity, carcinogenicity or disruption of endocrine and reproductive health, among others [15]. NEOs have emerged as a more environmentally and health-friendly alternative, and they are extensively used [16], although in vitro studies have shown that they could pose similar toxicities. According to the EFSA annual report and the common alerts found in the Rapid Alert System Feed and Food (RASFF), the compounds most detected in food samples belong to the mentioned compound families [17–19]. In this context, these compounds have also been the most detected pesticides in previous biomonitoring studies in Spain and other countries [5, 15, 20–22]. Likewise, in addition to food, which is the main route of exposure to pesticides, it has been observed that people who live near cultivation areas or farmers are highly exposed [23–26].

In addition to pesticides, mycotoxins are other chemicals of growing concern. Most RASFF alerts are related to the presence of mycotoxins in food [27]. Mycotoxins are secondary toxic metabolites naturally produced by several species of fungi, with *Aspergillus*, *Fusarium* and *Penicillium* being the predominant ones. Some of them are carcinogenic compounds (or suspected to be) and show a wide range of health effects such as hepatotoxic, nephrotoxic, cytotoxic, immunosuppressive, inflammatory, neurotoxic and estrogenic effects [28, 29]. Their occurrence in food occurs mainly, among others, in nuts, spices, cereals, wine or beer [30]. These compounds are normally produced under conditions of high temperature and humidity, which is of special concern in some Mediterranean countries such as Spain [6, 7, 27]. Among the more than 300 existing mycotoxins, aflatoxins, ochratoxins, zearalenone (ZEN) and *Fusarium* toxins (including trichothecenes and some emerging mycotoxins such as enniatins and beauvericin) are the most prevalent mycotoxins in food and feed and hence the most biomonitored ones [31–34].

Urine is the preferable biomonitoring matrix since it is a less invasive matrix that could be collected over long periods of time. In addition, urine is the main excretion route for environmental chemicals and metabolites [35]. Specifically, urine is the recommended matrix for biomonitoring mycotoxins and pesticides, as most of them or their metabolites are excreted in it [9, 15, 36, 37]. In this matter, solid-phase extraction (SPE) and miniaturised 96-well plate SPE, employing hydrophilic-lipophilic balance (HLB), graphitized carbon black (GCB), Strata X or C18 cartridges, have been widely used for the pre-concentration of analytes due to their typical low concentrations expected in urine samples [15, 21, 29, 34, 38–40].

Even though pesticides and mycotoxins represent a high percentage of the RASFF alerts and that there are some biomonitoring studies already published [41, 42], studies including both group of compounds are scarce. In this context, the aim of this work is the development of an analytical methodology employing targeted analysis to determine the co-exposure of the most commonly found pesticides and mycotoxins and their biomarkers of exposure in urine. The method has been fully validated and applied to analyse urine samples from people occupationally exposed to pesticides (farmers) and in the general population.

## Materials and methods

### Chemicals and instrumentation

Aflatoxin B1, aflatoxin B2, aflatoxin G1, aflatoxin G2, aflatoxin M1, deoxynivalenol (DON), 3-acetyl DON (3-AcDON), 15-acetyl DON (15-AcDON), deepoxydeoxynivalenol (DOM), ZEN, α-zearalenol (α-ZOL), β-zearalenol (β-ZOL), DON ^13^C15, ochratoxin α (OTα), ochratoxin A (OTA), ochratoxin B (OTB), enniatin A, A1, B and B1, beauvericin, T2-toxin and HT2-toxin were obtained from Techno Spec (Barcelona, Spain) and zearalanone (ZAN) from Toronto Research Chemicals (Ontario, Canada).

Clothianidin D3, permethrinic acid, denitro-imidacloprid, hydroxycarbendazim, clothianidin, 2-diethylamino-6-methyl-4-pyrimidinol (DEAMPY), tebuconazole, 3-phenoxybenzoic acid (PBA), imidacloprid-olefin, acetamiprid-desmethyl, dimethylphosphate (DMP), pirimiphos-methyl, azoxystrobin, dimethoate, diethylphosphate (DEP), cypermethrin, diethylthiophosphate (DETP), diethyldithiophosphate (DEDTP), tebuconazole-butylhydroxy, carbendazim D3, 3,5,6-trichloro-2-pyridinol (TCPY), clothianidin-desmethyl, acephate and azoxystrobin were purchased by LGC (Augsburg, Germany). Dimethylthiophosphate (DMTP) and dimethyldithiophosphate (DMDTP) were acquired from Cambridge isotope laboratories (Andover, MA, USA). Creatinine, creatinine D3, carbendazim, acetamiprid, imidacloprid, chlorpyrifos and chlorpyrifos methyl were supplied by Merck (Darmstadt, Germany).

All analytical standards had a purity greater than 97% (except ZEN, DMP, pirimiphos-methyl and clothianidin-desmethyl with purities greater than 95%). Individual standard solutions for each compound were prepared at 1000 mg/L by dissolving 1 mg of each solid standard in 1 mL of methanol. These solutions were used to prepare working standard solutions at concentrations of 100 and 10 mg/L. All solutions were kept frozen at − 20˚C.

β-Gluguronidase from *Helix pomatia* (100,000 units/mL) to perform the deconjugation of glucuronide metabolites was obtained from Merck.

An Agilent 1290 Infinity I System (Agilent Technologies, Santa Clara, CA, EEUU) with a Hypersil Gold aQ column (100 × 2.1 mm, 1.9 µm particle size), coupled to an API 3200 triple quadrupole (QqQ) mass spectrometer (AB Sciex; Darmstadt, Germany), was used for compound determination. More information on “Chemicals and Instrumentation” is provided in Supplementary Material.

### Compound selection

As briefly discussed above, exposure to mycotoxins and pesticides occurs primarily through food intake. Thereby, information on their occurrence in food was used to select the target compounds [5–7]. For the selection of pesticides, the last annual EFSA and FDA reports (2019 and 2020), as well as the most common RASFF alerts, were evaluated. These reports include the results of analyses for a large number of compounds (about 800 compounds) in several food products (180,000 food samples in the last EFSA report) [17–19, 27, 43]. Thus, 92 compounds were selected as the most important because they were the most frequently detected and/or exceeded the maximum residue level (MRL) to a greater extent. Among these compounds, non-amenable compounds by liquid chromatography (LC), i.e. gas chromatography (GC) compounds, were discarded, and representative compounds of different pesticide families were selected, specifically from the group of PY insecticides, NEO pesticides, fungicides and OP pesticides. The resulting list is shown in Table 1, named as “parent compounds”.

**Table 1:** Compounds of interest selected for their analysis in urine

Family	Parent compounds	Biomarker of exposure	Ref
Pesticides			
Pyrethroid insecticides	Cypermethrin	DCCA	[15, 46, 47]
		PBA	
Non-specific organophosphate pesticides	Chlorpyrifos-methyl, dimethoate and pirimiphos-methyl	DMP	
		DMTP	
		DMDTP	
	Chlorpyrifos	DEP	
		DETP	
		DEDTP	
Organophosphate pesticides	Chlorpyrifos and chlorpyrifos-methyl	TCPY	
	Pirimiphos-methyl	DEAMPY	
	Acephate	Acephate	
	Dimethoate	Dimethoate	
Neonicotinoid pesticides	Acetamiprid	Acetamiprid‐N‐desmethyl	[20, 48]
	Clothianidin	Clothianidin‐N‐desmethyl	
	Imidacloprid	Hydroxy-imidacloprid	
		Imidacloprid-olefin	
		Imidacloprid-guanidine	
Fungicides	Azoxystrobin	Azoxystrobin acid	[21]
	Carbendazim	Hydroxycarbendazim	[49]
	Tebuconazole	Hydroxytebuconazole	[50]
Mycotoxins			
Aflatoxins	Aflatoxin B1	Aflatoxin M1	[51]
	Aflatoxin B2		
	Aflatoxin G1		
	Aflatoxin G2		
Trichothecenes	DON	15-AcDON	[52]
		3-AcDON	
		DOM	
	T2 toxin		[53]
	HT2 toxin		
Zearalenones	ZEN	ZAN	[32, 40]
		α-ZOL	
		β-ZOL	
Ochratoxins	Ochratoxin A	Ochratoxin B	
		Ochratoxin α	
Emerging mycotoxins	Enniatin A		[34]
	Enniatin A1		
	Enniatin B		
	Enniatin B1		
	Beauvericin		

Abbreviation: *15-AcDON* 15-acetyldeoxynivalenol, *3-AcDON* 3-acetyldeoxynivalenol, *α-ZOL* α-zearalenol, *β-ZOL* β-zearalenol, *DCCA* cis-permethrinic acid, *DEAMPY* 2-diethylamino-6-methyl-4-pyrimidinol, *DEP* diethylphosphate, *DETP* diethylthiophosphate, *DEDTP* diethyldithiophosphate, *DMP* dimethylphosphate,, *DMTP* dimethylthiophosphate, *DMDTP* dimethyldithiophosphate, *DOM* deepoxy-deoxynivalenol, *DON* deoxynivalenol, *PBA* 3-phenoxybenzoic acid, *TCPY* 3,5,6-trichloro-2-pyridinol, *ZAN* zearalanone, *ZEN* zearalenone

For the selection of mycotoxins, the most commonly appeared in food and with the highest rate of positives in food samples were selected. In this matter, the selected compounds include aflatoxins (which present high toxicity, being the most legislated and the most reported in RASFF alerts [27]), trichothecenes (with a high occurrence in food samples highly consumed, such as cereals and nuts [4]), OTA, ZEN and related compounds (with a high detection rate (DR) in biological samples [33, 44]) and enniatins and beauvericin (which are emerging mycotoxins frequently detected in cereals [45]).

Once the pesticides and mycotoxins of interest were selected, their most common human metabolites were searched in bibliography. Table 1 includes those metabolites finally selected to be also covered by the developed method, named as “biomarkers of exposure”.

### Sample collection and preparation

The first step prior to sample collection was the development of a questionnaire to evaluate possible exposure sources of the participants in this study. The use of questionnaires is highly recommended in biomonitoring studies to eliminate possible co-exposure sources and to find out where an exposure might come from [54]. A homemade questionnaire was developed, which includes some important points to clarify whether living area, work environment or daily consumer habits could have some relationship with the exposure to pesticides and mycotoxins detected. The samples were collected in the University of Granada or in the University of Almeria, from people residing in different locations in the regions of Granada (Spain) (i.e. Granada, Calicasas and Cogollos Vega) and Almería (Spain) (i.e. Almería, Abla, El Ejido and Campohermoso), respectively. After completing the questionnaires, approximately 10-mL urine samples were collected in 15-mL polypropylene tubes and stored at − 20°C. Lower storage temperatures were not considered as most of the studied chemicals are stable for months at -20°C [55].

Frozen samples were thawed and let reached ambient temperature before extraction. Then, they were centrifuged to remove precipitates, and subsequently creatinine levels were measured to normalize chemical concentrations among samples. A previously published method was adapted to measure creatinine levels [56]. Briefly, 50 µL of urine were submitted to successive tenfold dilutions with water containing 0.1% (*v/v*) aqueous NH_3_ until reaching a 1:10,000 dilution. To the final dilution, 100 µL of creatinine-d3 at 1 mg/L was added prior chromatographic analysis (“Sample analysis” section). A standard calibration curve in the concentration range of 5 and 250 µg/L was used to determine creatinine.

After creatinine measurement, two protocols were followed. In one of them, samples were directly analysed after the extraction following a salting-out liquid–liquid extraction (SALLE) protocol. First, 50 µL of the standard deuterated solution, containing DON ^13^C15, clothianidin D3 and carbendazim D3 (1 mg/L), was added to 1 mL of urine in a 15-mL polypropylene tube. Then, 1 mL of acetonitrile was added, and, after stirring by vortex for 10 min, 0.8 g of ammonium sulphate was added. The mixture was vortexed again for 5 min, and the tubes were centrifuged at 9000 rpm (7690 rcf) for 10 min in a refrigerated centrifuge at 4˚C. The supernatant was transferred to a 4-mL glass vial and evaporated under a N_2_ stream. The solid residue was reconstituted in 100 µL of a mixture of methanol–water (50:50, *v/v*) containing 0.1% (*v/v*) formic acid. The resulting solution was transferred to a 0.5-mL Amicon ultra centrifugal filter (Merck) and centrifuged for 2 h at 12,000 rpm (10,250 rcf) at 4˚C. The final extract was transferred to a 100-µL glass insert and injected into the chromatographic system.

On the other hand, since urine contaminants are normally excreted in their conjugated forms (i.e. glucuronide or sulphate conjugates), deconjugation steps were required to measure the total amount of contaminants [35]. Thus, deconjugation was performed by acidifying the samples at pH 5.1 with 200 μL of a 1 M acetic acid‐ammonium acetate buffer solution and adding β‐glucuronidase at a concentration of 6000 units/mL (60 µL). This mixture was left during 12 h at 37˚C and then SALLE protocol was applied.

### Sample analysis

Samples were analysed by LC-QqQ tandem mass spectrometry (MS/MS) working in schedule multiple reaction monitoring (sMRM) (minimum 100 data points for peak). A Hypersil Gold aQ column thermostated at a temperature of 40˚C was employed for the chromatographic separation. The mobile phase consisted of water (solvent A) and methanol (solvent B), containing both solvents 0.2% (*v/v*) formic acid and 4 mM ammonium formate. A gradient programme for the mobile phase composition during the separation was established as follows. The composition gradient started with 10% of organic phase (B). This percentage was maintained for 1 min, and then it was increased until 50% in 4 min. After keeping it constant for 1 min, it was increased again at 90% of B in 5 min. It was kept at this percentage for 1 min. Initial conditions were recovered in 1 min, and the column was equilibrated for 3 min, resulting in a total analysis time of 16 min. Injection volume was set at 10 µL and flow rate at 0.3 mL/min.

The method of Fraselle et al. was used for the determination of creatinine with some differences [56]. Instead of an Acquity UPLC HSS T3 (2.1 × 100 mm, 1.8 µm) chromatographic column, the aforementioned Hypersil Gold aQ UPLC column was used to facilitate the simultaneous determination of biomarkers of interest and creatinine. The mobile phase consisted of water containing 0.1% (*v/v*) ammonium hydroxide solution (25%, *v/v*) (solvent A) and acetonitrile (solvent B). The elution gradient was the same as in the reported method [56].

## Results and discussion

### Extraction optimization

Taking into account the wide range of physicochemical properties of the selected compounds, it was necessary to apply non-exhaustive sample preparation methods to obtain satisfactory recoveries for all of them. In this matter, solid-phase extraction (SPE) and miniaturised 96-well plate SPE, employing hydrophilic-lipophilic balance (HLB), graphitized carbon black (GCB), Strata X or C18 cartridges, have been widely used for the pre-concentration of analytes due to their typical low concentrations expected in urine samples [15, 21, 29, 34, 38–40]. However, the proposed methodologies have been applied to the determination of a reduced number of compounds with similar physicochemical characteristics, making their application impossible when a larger number of compounds with different polarities are determined. In this case, the alternative is the application of a dilute-and-shoot protocol or the use of non-selective sample treatments such as liquid–liquid extraction (LLE).

In the context of this study, three sample treatments were tested: dilute-and-shoot, QuEChERS and SALLE procedures. Performance characteristics of the proposed analytical method using the 3 above-mentioned procedures are included in Table 2. For the dilute-and-shoot method, samples were subjected to a tenfold dilution with a mixture of methanol–water (50:50, *v/v*) containing 0.1% (*v/v*) formic acid. Although a simple sample preparation is always preferred, the limits of quantification (LOQs) obtained using this methodology were too high (i.e. ranging from 0.5 to 200 µg/L, Table 2), and considering that contaminant concentrations are expected to be low in urine samples, this approach was discarded [57].

**Table 2:** Performance characteristics of the proposed LC–MS method depending on the evaluated sample treatments

Compound	Dilute and shoot	QuEChERS^a^		SALLE (selected sample treatment)^a^							
		LOQs (µg/L)	Recoveries (%)^b^	LOQs (µg/L)	Recovery (%)			Intra-day/Inter-day precision (RSDs, %)			Matrix effect (%)
	LOQs (µg/L)		100 µg/L		1 µg/L	10 µg/L	100 µg/L	1 µg/L	10 µg/L	100 µg/L	
Pesticides											
5-hydroxycarbendazim	10	0.25	92 ± 7	0.1	89	94	95	14/11	16/17	5/11	− 42
Acephate	50	10	59 ± 15^c^	10	-	59	63	-	3/7	1/10	299
Acetamiprid	1	0.5	81 ± 9	0.25	94	87	75	13/17	14/19	8/7	− 58
Acetamiprid-desmethyl	25	5	92 ± 7	5	-	93	89	-	6/15	1/12	− 58
Azoxystrobin	1	0.5	90 ± 11	0.25	91	94	91	10/18	7/15	5/11	− 45
Azoxystrobin acid	2.5	0.5	94 ± 9	0.5	100	76	97	16/19	8/6	1/19	− 51
Carbendazim	5	2.5	97 ± 1	0.5	111	97	96	14/16	8/16	4/16	17
Chlorpyrifos	1	2.5	56 ± 18	0.5	69	81	74	7/13	11/15	6/11	− 21
Chlorpyrifos methyl	5	2.5	63 ± 17	2.5	-	85	93	8/11	8/14	5/16	− 20
Clothianidin	10	10	90 ± 10	10	-	93	88	13/22	12/14	2/17	87
Clothianidin desmethyl	200	50	82 ± 8	100	-	-	78	-	-	5/16	50
Cypermethrin	100	100	51 ± 21	50	-	-	81	-	-	18/18	131
DEAMPY	5	2.5	85 ± 11	1	98	88	83	10/17	11/14	19/17	− 63
DEDTP	200	50	44 ± 14	100	-	-	56	-	-	18/7	− 72
DEP	100	25	43 ± 16	25	-	-	56	-	-	13/15	− 57
DETP	200	50	40 ± 17	50	-	-	48	-	-	12/13	− 63
Desnitro-imidacloprid	25	10	70 ± 6	10	-	61	69	-	13/14	6/10	− 80
Dimethoate	2.5	0.5	79 ± 1	0.5	90	82	82	1/14	11/13	1/18	− 44
DMDTP	100	50	40 ± 15	50	-	-	66	-	-	7/8	− 94
DMP	200	50	42 ± 13	100	-	-	50	-	-	15/5	− 88
DMTP	200	25	46 ± 18	10	-	58	62	-	6/19	10/11	− 98
Imidacloprid	5	1	86 ± 3	1	85	96	85	15/13	3/16	4/17	− 37
Imidacloprid olefin	100	25	93 ± 6	25	-	-	90	-	-	6/16	− 43
Hydroxy-imidacloprid	10	2.5	92 ± 2	2.5	-	71	88	-	9/8	3/17	51
PBA	10	2.5	90 ± 7	1	72	80	89	8/19	16/14	3/15	− 75
DCCA	25	2.5	89 ± 4	2.5	-	88	81	-	14/18	6/19	− 19
Pirimiphos methyl	5	0.5	73 ± 8	0.1	73	62	66	17/14	6/14	4/19	− 16
TCPY	10	2.5	86 ± 4	2.5	-	90	87	-	17/19	6/19	75
Tebuconazole	1	0.5	65 ± 15	0.1	83	76	92	13/13	6/15	7/16	1
Tebuconazole hydroxy	5	1	85 ± 12	0.5	87	82	99	6/14	7/14	4/7	− 11
Mycotoxins											
15-AcDON	25	5	85 ± 9	5	-	86	87	-	16/18	10/14	− 43
3-AcDON	25	5	91 ± 7	2.5	-	97	92	-	6/17	18/11	− 54
Aflatoxin B1	1	0.25	86 ± 2	0.25	83	82	83	10/10	14/7	5/15	− 67
Aflatoxin B2	5	0.5	75 ± 4	0.5	92	77	73	11/11	1/8	2/9	− 59
Aflatoxin G1	2.5	0.5	79 ± 4	0.5	93	81	80	19/17	10/6	9/8	− 61
Aflatoxin G2	5	0.5	77 ± 3	0.5	101	85	72	18/6	7/7	7/6	− 71
Aflatoxin M1	1	0.25	79 ± 8	0.1	88	88	78	11/9	10/13	10/8	− 55
α-ZOL	5	1	94 ± 10	1	63	84	96	9/14	7/5	5/19	22
Beauvericin	10	0.25	69 ± 16	0.1	85	88	74	12/17	13/16	9/14	− 28
β-ZOL	5	2.5	95 ± 12	2.5	-	79	86	-	16/14	7/19	8
DOM	100	1	91 ± 5	1	72	85	90	12/17	13/14	4/16	− 48
DON	50	5	89 ± 7	5	-	95	81	-	14/14	6/13	39
Enniatin A	1	0.25	61 ± 13	0.1	76	85	71	16/12	10/15	15/9	− 22
Enniatin A1	0.5	0.25	59 ± 17	0.1	77	79	73	7/14	12/11	17/4	− 19
Enniatin B	0.5	0.25	65 ± 19	0.1	95	72	76	10/16	12/5	10/9	− 23
Enniatin B1	0.5	0.25	66 ± 18	0.1	78	84	71	13/17	13/11	5/8	− 18
HT2 toxin	25	2.5	62 ± 16	5	-	58	55	-	10/13	6/19	− 87
Ochratoxin A	2.5	0.5	83 ± 8	0.5	94	81	80	6/11	9/13	6/17	− 30
Ochratoxin α	10	0.5	78 ± 4	1	64	90	74	8/12	7/14	1/8	− 9
Ochratoxin B	0.5	0.25	88 ± 6	0.1	84	81	85	18/18	9/13	5/15	− 35
T2 toxin	10	2.5	60 ± 18	2.5	-	58	56	-	14/10	7/11	− 69
ZAN	10	2.5	88 ± 6	2.5	-	73	87	-	5/6	4/19	24
ZEN	1	1	96 ± 9	0.5	95	72	91	12/19	3/12	3/19	− 15

^a^ Number of replicates (*n*) = 5

^b^ RSD with *n* = 5

^c^ Validation parameters outranging adequate values (recoveries < 60% or RSD > 20%) are highlighted in red

Abbreviation: *15-AcDON* 15-acetyldeoxinivalenol, *3-AcDON* 3-acetyldeoxinivalenol, *α-ZOL* α-zearalenol, *β-ZOL* β-zearalenol, *DCCA* cis-permethrinic acid, *DEAMPY* 2-diethylamino-6-methyl-4-pyrimidinol, *DEDTP* diethyl dithiophosphate, *DEP* diethyl phosphate, *DETP* diethyl thiophosphate, *DMDTP* dimethyl dithiophosphate, *DMP* dimethyl phosphate, *DMTP* dimethyl thiophosphate, *DOM* deepoxy-deoxynivalenol, *DON* deoxinivalenol, *PBA* 3-phenoxybenzoic acid, *TCPY* 3,5,6-trichloro-2-pyridinol, *ZAN* zearalanone, *ZEN* zearalenone

Thus, a QuEChERS-based methodology already employed to determine a small number of mycotoxins or pesticides in urine was tested [31, 44, 58–61]. Similarly to these studies, 1 mL of urine was extracted with 1 mL of acetonitrile and assisted by vortex agitation for 10 min. Then, 0.8 g of MgSO_4_ (phase separation salt) and 0.1 g C18 (dispersive-SPE (dSPE) sorbent) were added. The mixture was vortexed for 5 min and centrifuged at 9000 rpm (7690 rcf) for 10 min at 4˚C, observing a liquid–liquid phase separation. The upper organic phase was collected, filtered through a 0.22-µm nylon filter (Agilent Technologies) and injected into the chromatographic system. Although LOQs were significantly better than those obtained with the dilute-and-shoot approach, extraction recoveries were low for some compounds (< 60%) when this methodology was applied (Table 2). Specifically, low extraction recoveries were observed for highly polar compounds, such as dialkyl phosphate metabolites (DAPs), and nonpolar compounds (i.e. GC amenable pesticides) such as chlorpyrifos, cypermethrin and tebuconazole, as well as enniatins and beauvericin. Low recoveries for highly polar compounds could be explained because they remain in the aqueous phase after the phase separation step, while nonpolar compounds could be adsorbed by the C18 sorbent during the dSPE step.

Simultaneously to QuEChERS extraction, the SALLE method was evaluated. In this case, 1 mL of urine was extracted with 1 mL of acetonitrile by vortexed for 10 min. Then, 0.8 g of ammonium sulphate were added to achieve the phase separation. The mixture was vortexed for 5 min and then centrifuged at 9000 rpm (7690 rcf) for 10 min at 4˚C. The organic phase was subsequently collected, filtered and injected into the chromatographic system. The recoveries for both polar and nonpolar compounds were better than those obtained by the QuEChERS procedure. However, the obtained LOQs were around twofold higher. For that reason, the acetonitrile phase was evaporated under nitrogen flow, and the residue was reconstituted with 100 µL of methanol–water 50:50 (*v/v*) containing 0.1% formic acid. As can be seen in Table 2, the LOQs were similar or slightly better than those obtained with the QuEChERS method, whereas extraction recoveries ranged between 60 and 130%, except for some DAPs that were lower than 60% but in any case better than QuEChERS recoveries. Thus, SALLE was selected as sample treatment.

### LC–MS/MS method optimization

Electrospray (ESI) MS parameters were optimized by individual infusion of all target compounds into the mass spectrometer at a concentration of 10 mg/L and prepared in a mixture of methanol–water (50:50, *v/v*) containing 0.1% (*v/v*) formic acid. Compounds were detected employing positive and negative ionization modes (see Supplementary material for more information about instrumentation). Precursor and product ions, as well as the collision energy (CE), the cell entrance potential (CEP), the collision cell exit potential (CXP), declustering potential (DP) and the entrance potential (EP), were optimized during this step. Optimal parameters were selected in order to obtain the highest sensitivity for the transitions selected for each compound (Table S1).

After ESI–MS characterization, the chromatographic separation was optimized. Different LC columns commonly used in the determination of chemical contaminants and residues were evaluated by analysing a standard mixture prepared in a urine blank at 100 µg/L. As no blank certified reference materials was available, samples with undetected levels of target compounds were chosen as “urine blank”. A generic mobile phase composed of water with ammonium formate (4 mM) (solvent A) and methanol (solvent B), both containing 0.2% (*v/v*) formic acid, was used to compare all the tested columns (Table 3), with a flow rate of 0.3 mL/min. In this sense, two elution gradients were also evaluated. In gradient 1, the starting conditions were established at 10% of organic phase (B). This percentage was maintained for 1 min, and then the percentage of the organic phase was increased to 90% in 10 min. After 1 min, the initial conditions were recovered in 1 min, and the column was equilibrated for 3 min. Gradient 2 was also started at 10% of B, holding it for 1 min. Then, it was increased to 50% of B in 4 min and held constant for 1 min. It was then increased to 90% of B in 5 min and held constant for 1 min. Initial conditions were recovered in 1 min, and the column was equilibrated for 3 min. Both elution gradients involved a total run time of 16 min. Gradient 2 offered the best performance for all the tested columns, so results commented below correspond to this elution gradient.

**Table 3:** Characteristic of the chromatographic columns tested

Column	Dimensions	Composition	Void volume (mL)	Void time (min)
Zorbax Eclipse Plus	100 × 2.1 mm, 1.8 µm particle size	C18	0.24	0.8
Zorbax Eclipse Plus	50 × 2.1 mm, 1.8 µm particle size	C18	0.12	0.4
Hypersil Gold	100 × 2.1 mm, 1.9 µm particle size	C18	0.24	0.8
Acquity HSS T3	150 × 2.1 mm, 1.8 µm particle size	C18	0.37	1.2
Cosmocore 2.6 PBr	100 × 2.1 mm, 2.6 µm particle size	Pentabromobenzyl	0.24	0.6
Hypersil Gold aQ	100 × 2.1 mm, 1.9 µm particle size	Polar endcapped C18	0.24	0.8

First, two Zorbax Eclipse Plus C18 columns (1.8 µm particle size) of different dimensions (100 × 2.1 mm and 50 × 2.1 mm) and one Hypersil Gold C18 column (100 × 2.1 mm, 1.9 µm particle size) were evaluated. These columns provided a relatively good separation for compounds of medium polarity. However, some high polarity compounds (i.e. DAPs metabolites) were not retained, eluting just after void time (0.8 min), whereas nonpolar compounds (i.e. chlorpyrifos, enniatins, beauvericin and cypermethrin) were highly retained in the columns and appeared as ghost peaks in the successive injections (Figs. S1.1 and S1.2).

To solve these problems, two other columns were employed. First, the Acquity UPLC HSS T3 column (150 × 2.1 mm, 1.8 µm particle size) was evaluated. This column has the ability to work under elution gradients with 100% of aqueous phase and, due to its longer dimension, is recommended to perform the separation of compounds with a broad range of properties. Thus, gradient 2, but starting it with 5% of B instead 10%, was used. Although polar compounds seemed to be better retained (only DMP and DMDTP appeared near to the void time of 1.2 min), some nonpolar compounds (chlorpyrifos, enniatin B, enniatin B1 and beauvericin) were strongly retained, having the same problem than with previous columns (Figs. S2.1 and S2.2). In addition, a Cosmocore 2.6PBr column (100 × 2.1 mm, 2.6 µm particle size) was also evaluated. This column was previously selected by Periat et al. as the best among 29 columns to perform the separation of a large list of compounds with different physicochemical properties [62]. To provide a better comparison between the different columns, the flow rate of the mobile phase was increased from 0.3 to 0.4 mL/min when using the Cosmocore 2.6PBr column, since the particle size in this column is larger. Results showed that performance was worse than with the previously evaluated columns. Although any polar compounds appeared in the void time (0.6 min), many of them were poorly retained, eluting 20 of them in the first 2 min. Besides, nonpolar compounds (i.e. enniatins and beauvericin) presented the same problem observed with the other tested columns (Figs. S3.1 and S3.2).

With the aforementioned columns, several medium polarity compounds co-eluted and some peak shapes were asymmetric and too wide (e.g. OTA in Zorbax Eclipse Plus column, DAPs in Acquity HSS T3 column or OTα and 15-AcDON in Cosmocore 2.6PBr column). Although performance for medium polarity and nonpolar compound could be improved by extending the elution gradient, in targeted methods, and specially in biomonitoring studies, time analysis needs to be as short as possible with the aim of being efficient in the number of samples analysed, so this possibility was discarded.

**Fig. 1:**
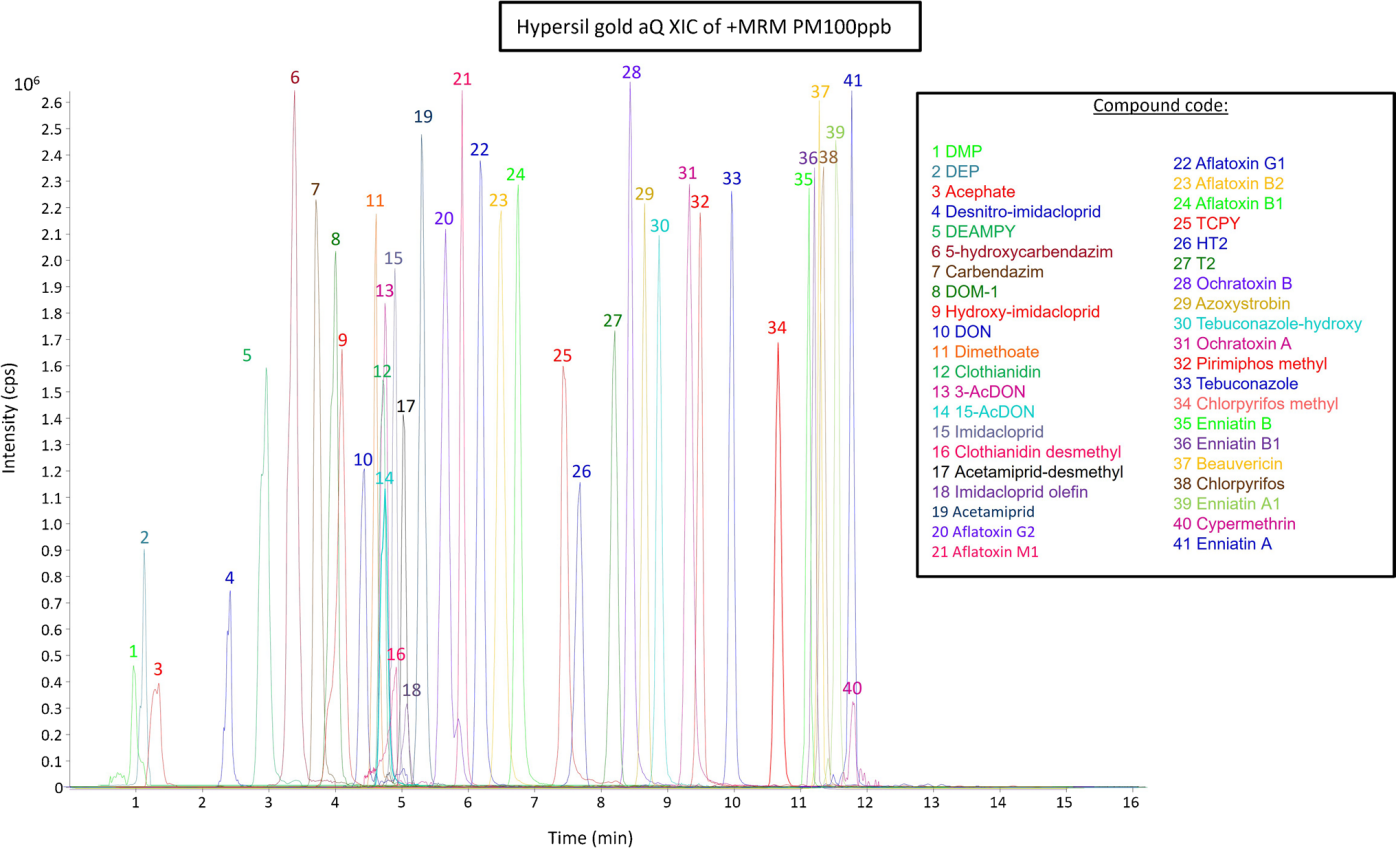
Extracted ion chromatogram of studied compounds (MRM in positive mode) at 100 µg/L

**Fig. 2:**
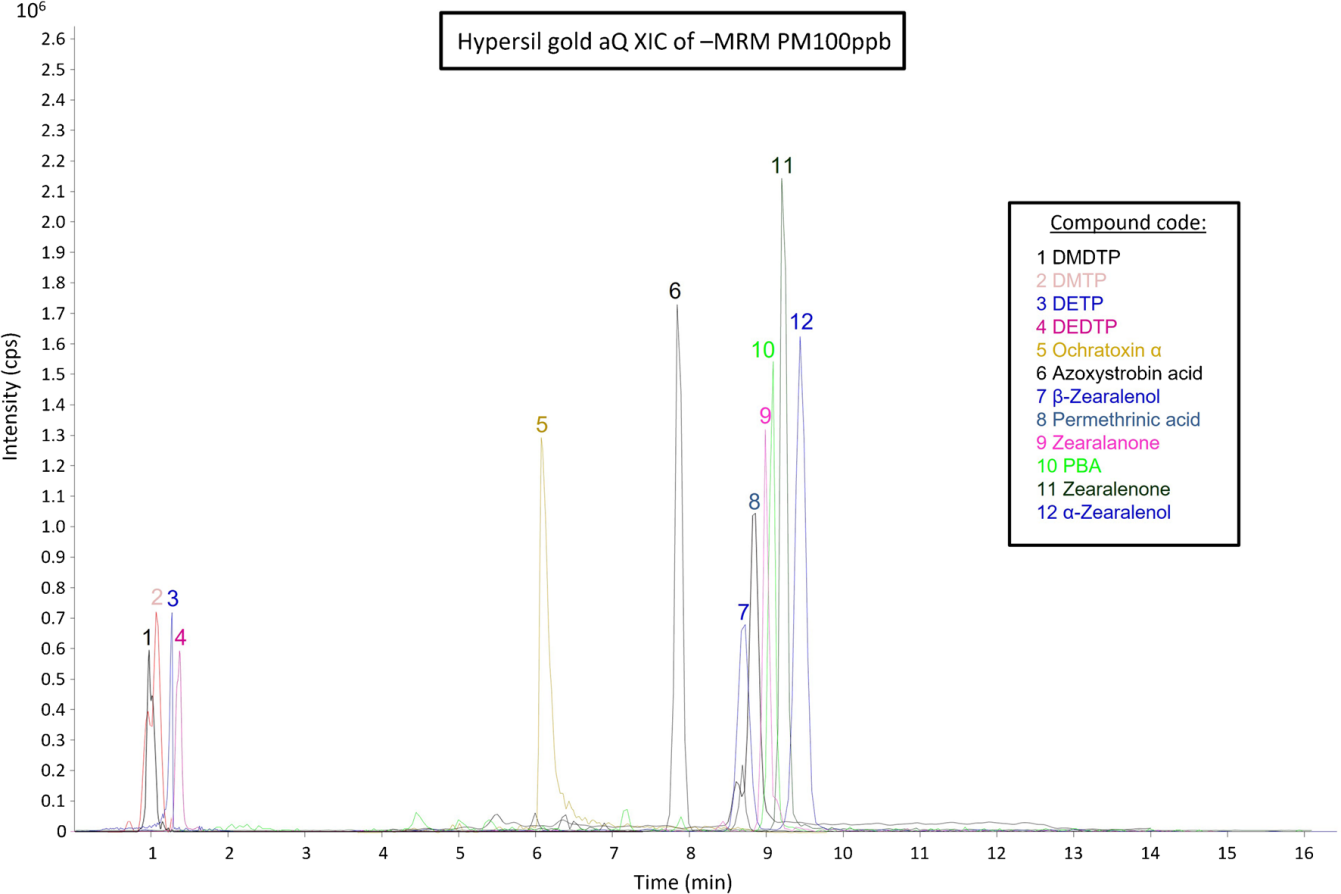
Extracted ion chromatogram of studied compounds (MRM in negative mode) at 100 µg/L

Finally, the polar endcapped Hypersil Gold aQ column (100 × 2.1 mm, 1.9 µm particle size) was evaluated. This column is designed to achieve separations of compounds with a wide range of physicochemical properties. Similar retention times were obtained for polar compounds using this column with respect to previous columns (i.e. elution times between 1 and 1.5 min and void time of 0.8 min). Nevertheless, considering that the analysis of DAPs requires specific analytical methodologies and that nonpolar compounds eluted before column re-equilibration (before 12 min), this was the finally selected column. Besides, all the compounds were scattered throughout the chromatogram, most of them with higher sensitivities and more symmetric peak shapes than with previous columns) (Figs. 1 and 2, corresponding to the compounds detected in positive and negative ionization modes, respectively).

### Method validation

SANTE guideline was followed for the method validation [63]. Although this guide is designed for the determination of pesticides in food samples, their parameter values could be employed for the validation of any analytical method including different matrices. Thus, different parameters such as specificity, linear and working range, precision, accuracy, limits of detection (LODs), LOQs and matrix effect were evaluated for method validation.

For specificity, endogenous compounds in urine were evaluated as interferences from the compounds of interest. Specificity was evaluated by comparison of extracted ion chromatograms (EICs) from blank and spiked blank samples, selecting four different urine blank samples. Although no signals were observed in the EICs for most ion transitions, signals were observed in seven of them (highlighted in red in Table S1) when urine blank samples were analysed. However, they appeared at different retention times than the target analytes and at a very low intensity. Therefore, satisfactory specificity was attributed to the developed method.

Linear working range was evaluated with matrix-matched calibration curves. At least six concentration levels were selected for the preparation of the calibration curves. The linear range covered from LODs to 250 µg/L, although accuracy and precision were only evaluated between LOQs and 100 µg/L, so this was the working range selected. Matrix effect was calculated by comparing standard and matrix-matched calibration curves, applying Eq. (1):

1 $$Matrix\;effect\left(\%\right)=\left(\frac{Matrix-matched\;calibration\;curve\;slope}{Solvent\;calibration\;curve\;slope}-1\right)\times100$$

As can be seen in Table 2, although some compounds presented not-significant matrix effects (i.e. values between − 20 and 20%), most of them require a matrix-matched calibration curve for their quantification in samples.

Trueness was evaluated in terms of apparent recoveries, which was assessed at three concentration levels, 1, 10 and 100 µg/L. They were studied by the analysis of five replicates for each fortification level. Trueness was considered acceptable if recoveries were between 60 and 130%, since exceptionally, recoveries above 60% and below 130% were accepted if precision values were lower than 20%. All the recoveries were satisfactory, with some exceptions such as acephate, some DAP metabolites and HT-2 and T-2 toxins (Table 2), for which precision values were acceptable.

Recoveries trials were performed in five non-consecutive different days for each concentration level in order to evaluate the inter-day precisions [relative standard deviations (RSDs, %)]. RSDs were acceptable if they were between 0 and 20%. Intra- and inter-day precision was acceptable for all the compounds, except for azoxystrobin acid at the lowest level (Table 2).

Finally, LOD and LOQs were established as the minimum detectable and quantifiable concentrations for each compound, respectively. They were set considering a minimal signal/noise ratio (*S/N*) of 3 for LOD and 10 for LOQ, providing acceptable recoveries and RSDs values in the case of LOQs. LODs ranged from 0.01 to 25 µg/L, whereas LOQs were lower than 5 µg/L for the most of compounds (78% of compounds).

### Urine sample analysis

Finally, urine samples from different volunteers were analysed to evaluate the applicability of the developed method. A total of 45 samples were subjected to this analytical methodology (Tables S2 and S3). Samples were divided according to occupation [i.e. farmers (*n* = 22) vs general population (*n* = 23)] and diet. In the case of diet, two splits were done: for mycotoxins, a high consumption (*n* = 17) *vs* low consumption (*n* = 28) of cereals and nuts was considered, since they are the most contaminated food with these compounds (mycotoxins found in fruits and vegetables are mainly alternaria mycotoxins and patulin, which are not monitored in this work [64]). On the other hand, for pesticides, a high consumption (*n* = 15) *vs* low consumption (*n* = 30) group of fruits and vegetables was selected, because they are the matrices with a higher incidence in these compounds [17–19]. Differences between groups were statistically evaluated using SIMCA (v17). Principal component analysis (PCA) was performed to assess an overview and determine if differences existed between groups. If groups were well differentiated (*R*^2^ ≥ 0.7), orthogonal partial least squares-discriminant analysis (OPLS-DA) and variable importance in projection (VIP) were used to distinguish the overall differences among datasets and explain the features that make them different. In addition, samples with and without enzymatic treatment were analysed to determine both glucuronide and free compounds.

All the samples except four were positive in at least one of the target compounds. Differences due to exposure to pesticides and mycotoxins were evaluated separately.

*Exposure to pesticides*. Pesticides with the highest DR were OPs and DAPs compounds, which were detected in 82% of the samples. Concentrations of these compounds were up to 40.1 µg/g creatinine (for DMTP). Chlorpyrifos, chlorpyrifos methyl and TCPY (its major metabolite), as well as DEAMPY (the major metabolite of pirimiphos-methyl), were the most detected OPs. These results are in concordance with previously reported studies [65]. PBA, a metabolite of PY insecticide exposure, was also detected in a high percentage of samples (42%), as previously reported [46], and with concentrations ranging from 6.9 to 58.7 µg/g creatinine.

Neither occupational exposure nor fruit and vegetable consumption seem to produce a significant difference between groups (Figs. S4 and S5). If only NEOs and fungicides are considered, DR for the general population group was lower (35%) compared to the farmer group (86%). Concentrations in the farmer group were also higher (up to 37.4 µg/g creatinine) than in the general population group (up to 1.8 µg/g creatinine), highlighting a higher exposure of farmers to these compounds. Indeed, on the days of sample collection, some farmers had applied phytosanitary products that include some of the monitored compounds. For example, subject of the sample 4 was in direct contact with Luna Experience product, which contains tebuconazole and fluopyram; the subject 8 used Navaron product, containing azoxystrobin, and Fasthrin 15 WG product, including cypermethrin; subjects 13, 15 and 16 applied Mospilan product, with acetamiprid; and subject 20 applied Perfekthion, with dimethoate. In addition, samples 17 and 18 are from agricultural technical experts who work in the same greenhouse as the farmer identified as subject 8. Although some differences seem to be observed between groups, no statistical differences were obtained when only NEO and PYs were considered as variables (Fig. S6). Regarding samples from the general population, only sample 31 has unusually high concentration levels of DCCA, PBA and 5-hydroxy imidacloprid. At this regard, this subject had used the ointment called Sarcop, which is an antiparasitic product that acts against the scabies mite and contains permethrin and an antiparasitic containing imidacloprid which is used in dogs to prevent the infection of fleas and ticks.

*Exposure to mycotoxins*. For mycotoxins, OTA (51% of the samples) and DON and its metabolites (33% of the samples) were the most detected compounds in both groups, in agreement with previous studies [66, 67]. While OTA levels were up to 8.9 µg/g creatinine, DON and its metabolites resulted in higher concentrations, up to 86.0 µg/g creatinine for DON glucuronide, being this metabolite the most detected. Furthermore, 15-AcDON and 3-AcDON were also detected at lower concentration levels (5.7 µg/g creatinine), and DOM was found at a maximum concentration of 9.3 µg/g creatinine. The detection of DOM in biological samples is very controversial. Although some authors have mentioned that this metabolite is not found in humans or it is formed at very low concentration levels to be detected [68], other studies have reported its presence in human urine samples [32, 44, 69]. In addition, other mycotoxins were also found in urine samples, such as ZEN and its metabolites (i.e. α-ZOL and ZAN), with DR of 26% and concentration levels between the LOQ and 46.9 µg/g creatinine, and emerging mycotoxins, namely enniatins (enniatin A1, B and B1) and beauvericin, with DR of 24% and concentration levels up to 1.2 µg/g creatinine. Of particular concern is the subject related to sample 9, as a concentration of α-ZOL of 46.9 µg/g creatinine was found, and this mycotoxin is considered a strong estrogenic compound [53].

**Fig. 3:**
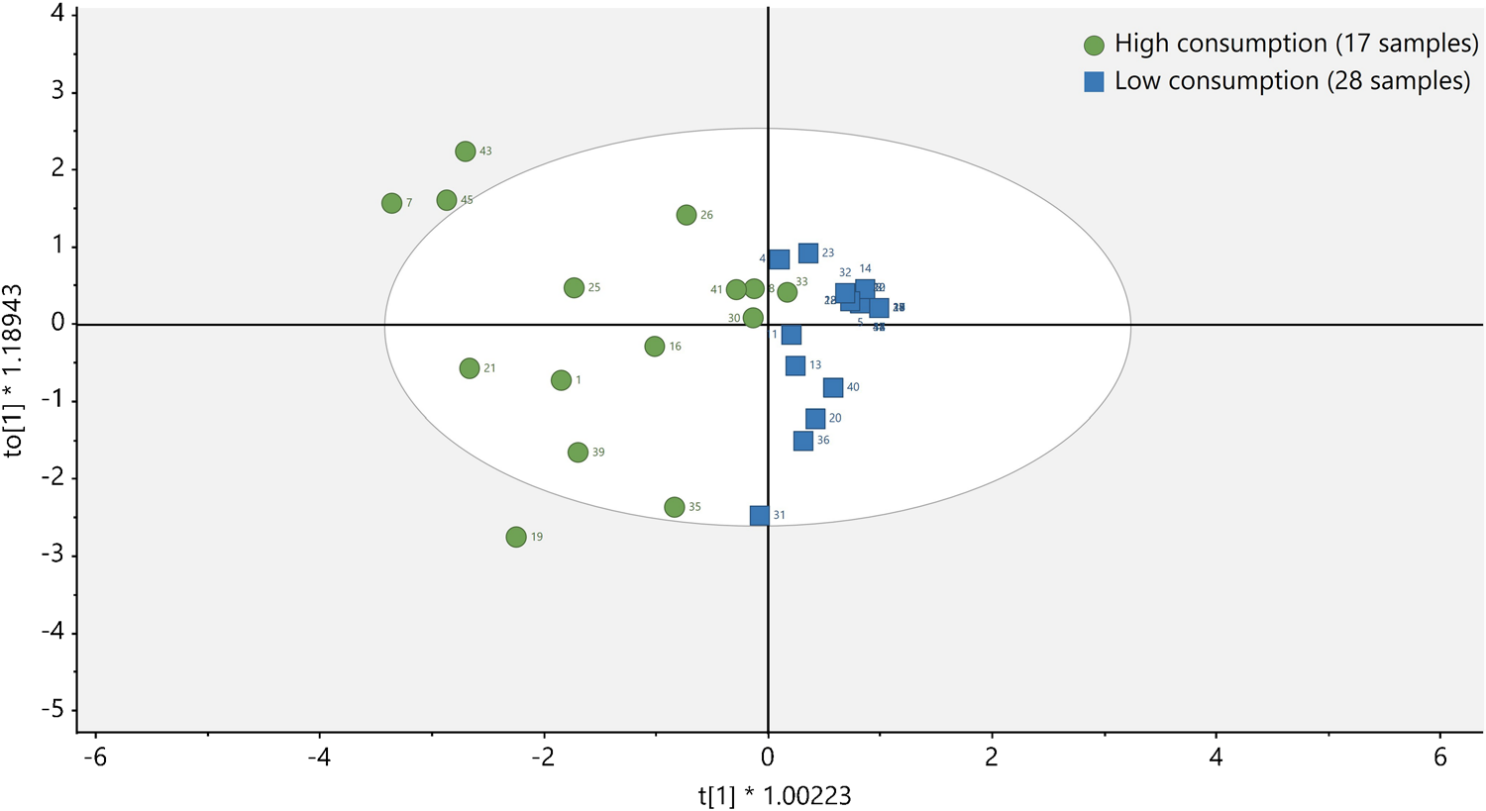
OPLS-DA for mycotoxins regarding high *vs* low consumption of cereals and nuts

Differences between occupational exposure and diet groups were also studied for mycotoxins. Although non-statistical differences were observed for occupation groups (Fig. S7), statistical differences were observed between groups with a high/low consumption of cereals and/or nuts. After the elimination of samples 6 and 9 which appeared as outliers (probably due to the high concentration of DON glucuronide found in these samples), the statistical model shown in Fig. S8 provided a quite good group separation (except for sample 11) with a *R*^2^ of 0.693. Over this model, OPLS-DA was performed, obtaining a *R*^2^ of 0.798 (Fig. 3). VIP showed that DON glucuronide, OTA and DOM were the compounds with a higher influence in the group separation (Fig. S9). Similar conclusions were achieved by other studies, such as the study of Penczynski et al. [67].

## Conclusion

In this study a new LC–MS for the determination of pesticides and mycotoxins was developed and validated in urine, with the aim to be applied in human biomonitoring studies. A SALLE method was optimized for the extraction of 30 pesticides (12 parent compounds and 18 urinary metabolites) and 23 mycotoxins (14 parent compounds and 9 urinary metabolites). Then, samples were analysed by UHPLC-QqQ-MS/MS. Method was then fully validated obtaining recoveries higher than 60% for most compounds and precision values lower than 20%.

Finally, the method was applied to the analysis of 45 urine samples from the southeast of Spain. Except for 4 samples, at least one compound was detected in all the samples, highlighting the need of deeper toxicological studies for biomonitoring of co-exposure to these substances. While all subjects seemed to be highly exposed to DAPs and pyrethroid metabolites, farmers seemed to be more exposed to NEO pesticides and PY (DR of 86%) than the general population (DR of 35%), although statistical differences were not found. On the other hand, mycotoxin exposure is high, especially for OTA and DON, being this exposure significantly higher in people with a higher consumption of cereals and/or nuts.

### Supplementary Information

Below is the link to the electronic supplementary material.

Supplementary file1 (DOCX 1496 KB)
