# Supplementary material for: Assessing human exposure to pesticides and mycotoxins: optimization and validation of a method for multianalyte determination in urine samples
Source: Anal Bioanal Chem. 2024 Feb 7;416(8):1935–49. doi: 10.1007/s00216-024-05191-2 (PMC10901940; doi:10.1007/s00216-024-05191-2)
Supplement: Supplementary file 1 — Supplementary file1 (DOCX 1496 KB) [file 216_2024_5191_MOESM1_ESM.docx]

**ANALYTICAL AND BIOANALYTICAL CHEMISTRY**

**Assessing Human Exposure to Pesticides and Mycotoxins: Optimization and validation of a method for multianalyte determination in urine samples**

Jesús Marín-Sáez^1,2,^*, Maykel Hernández-Mesa^1^, Jose A. Gallardo-Ramos^3^, Laura Gámiz-Gracia^1^, Ana M. García-Campaña^1,^*

^1^Department of Analytical Chemistry, Faculty of Sciences, University of Granada, Campus Fuentenueva s/n, E-18071 Granada, Spain

^2^Research Group “Analytical Chemistry of Contaminants”, Department of Chemistry and Physics, Research Centre for Mediterranean Intensive Agrosystems and Agri-Food Biotechnology (CIAIMBITAL), University of Almeria, Agrifood Campus of International Excellence, ceiA3, E-04120, Almeria, Spain

^3^ Department of Food Technology, Engineering and Science. Applied Mycology group, AGROTECNIO-CERCA Center, University of Lleida, 25198 Lleida, Spain

ORCID CODES

Jesús Marín-Sáez: 0000-0002-4153-9788

Maykel Hernández Mesa: 0000-0002-8308-526X

Laura Gámiz-Gracia: 0000-0002-8880-5000

Ana M. García-Campaña: 0000-0002-3191-3350

*Corresponding authors: J. Marín Sáez ([jms485@ual.es](mailto:jms485@ual.es)); A.M. García-Campaña ([amgarcia@ugr.es](mailto:amgarcia@ugr.es))

**Instrumental information**

For the extraction step a multi-tube vortexer model BV1010 from Benchmark Scientific (Sayreville, NJ, USA), a Universal 320R centrifuge from Hettich Zentrifugen (Tuttlingen, Germany) and a nitrogen dryer EVA-EC System from VLM GmbH (Bielefeld, Germany) were used.

An Agilent 1290 Infinity I System (Agilent Technologies; Waldbronn, Germany) equipped with a quaternary pump, a degasser, an autosampler (with 20-μL injection loop) and a column thermostat was used for the chromatographic separation. A Hypersil Gold aQ column (100 x 2.1 mm, 1.9 µm particle size) provided by Thermo Fisher Scientific (Les Ulis, France) was used for the separation. The liquid chromatography (LC) system was coupled to an API 3200 triple quadrupole (QqQ) mass spectrometer (AB Sciex; Darmstadt, Germany). The system was equipped with a Turbo V electrospray ionization (ESI) source at temperature of 600°C. Ion spray voltage was set at 5250 V whereas nebulizing and drying gases (nitrogen) and curtain gas (nitrogen) were set at 50 psi (344.7 kPa) and 40 psi (275.8 kPa) respectively.

MS/MS experiments were performed using schedule multiple reaction monitoring (sMRM) mode with an acquisition window of ±30 s, a minimum of 100 data points per peak and employing nitrogen as collision gas (5 psi, 34.5 kPa). At least one precursor ion and two product ions were monitored for all analytes except for 3-Phenoxybenzoic acid. Declustering potential (DP), entrance potential (EP), collision entrance potential (CEP), collision energy (CE) and collision cell exit potential (CXP) where optimized for all the monitored compounds (Table S1).

Instrumental data were collected and processed by the Analyst® Software (version 1.5) using the Scheduled MRM™ Algorithm (AB SCIEX).

**Table S1:** ESI-MS parameters for the studied compounds

| Compound | Retention time (min) | Adduct | Precursor ion | DP | EP | CEP | Product ions* | CE | CXP |
| --- | --- | --- | --- | --- | --- | --- | --- | --- | --- |
| Creatinine | 1.75 | [M+H]^+^ | 114.1 | 9 | 11 | 8 | **86.1** | **15** | **4** |
|  |  |  |  |  |  |  | 44.1 | 15 | 4 |
| Creatinine-D3 | 1.75 | [M+H]^+^ | 117.1 | 25 | 10 | 12 | **89.1** | **20** | **6** |
|  |  |  |  |  |  |  | 47.0 | 30 | 6 |
| DMP | 0.95 | [M+H]^+^ | 127.2 | 45 | 12 | 10 | **94.9** | **15** | **3** |
|  |  |  |  |  |  |  | 79.0 | 30 | 3 |
| DMDTP | 0.99 | [M-H]^-^ | 156.8 | -75 | -3 | -20 | 141.6 | -15 | -9 |
|  |  |  |  |  |  |  | **112.0** | **-30** | **-9** |
| DMTP | 1.05 | [M-H]^-^ | 141.0 | -45 | -3 | -10 | 96.0 | -45 | -9 |
|  |  |  |  |  |  |  | **125.7** | **-15** | **-9** |
| DEP | 1.25 | [M+H]^+^ | 155.0 | 45 | 6 | 10 | **98.9** | **15** | **9** |
|  |  |  |  |  |  |  | 81.0 | 45 | 9 |
| DETP | 1.25 | [M-H]^-^ | 169.0 | -45 | -6 | -10 | **95.0** | **-15** | **-9** |
|  |  |  |  |  |  |  | 141.0 | **-30** | **-9** |
| DEDTP | 1.35 | [M-H]^-^ | 185.0 | -45 | -6 | -10 | **111.0** | **-15** | **-9** |
|  |  |  |  |  |  |  | 157.0 | -30 | -9 |
| Acephate | 1.50 | [M+H]^+^ | 184.1 | 30 | 3 | 10 | **142.8** | **15** | **3** |
|  |  |  |  |  |  |  | 125.0 | 30 | 6 |
| Desnitro-imidacloprid | 2.53 | [M+H]^+^ | 211.1 | 75 | 3 | 20 | **125.9** | **45** | **9** |
|  |  |  |  |  |  |  | 133.1 | 30 | 9 |
| DEAMPY | 3.09 | [M+H]^+^ | 182.2 | 75 | 3 | 10 | 137.1 | 30 | 6 |
|  |  |  |  |  |  |  | 126.1 | 30 | 6 |
|  |  |  |  |  |  |  | **154.0** | **30** | **6** |
| 5-Hydroxycarbendazim | 3.48 | [M+H]^+^ | 208.1 | 60 | 3 | 10 | **176.2** | **45** | **9** |
|  |  |  |  |  |  |  | 208.0 | 5 | 9 |
| Carbendazim-D3 | 3.79 | [M+H]^+^ | 195.2 | 45 | 3 | 20 | **132.1** | **45** | **9** |
|  |  |  |  |  |  |  | 160.3 | 30 | 6 |
| Carbendazim | 3.81 | [M+H]^+^ | 192.2 | 45 | 9 | 20 | **159.9** | **45** | **9** |
|  |  |  |  |  |  |  | 132.2 | 45 | 6 |
| Deepoxy-deoxynivalenol | 4.02 | [M+H]^+^ | 281.0 | 30 | 3 | 10 | 189.0 | 30 | 6 |
|  |  |  |  |  |  |  | 187.1 | 30 | 6 |
|  |  |  |  |  |  |  | **215.1** | **30** | **6** |
| Hydroxy-imidacloprid | 4.1 | [M+H]^+^ | 272.1 | 60 | 3 | 20 | **225.4** | **30** | **6** |
|  |  |  |  |  |  |  | 191.4 | 30 | 3 |
| Clothianidin D3 | 4.58 | [M+H]^+^ | 253.3 | 60 | 3 | 30 | **132.2** | **45** | **9** |
|  |  |  |  |  |  |  | 172.0 | 30 | 9 |
| Deoxynivalenol | 4.63 | [M+H]^+^ | 296.9 | 60 | 3 | 20 | **175.3** | **30** | **6** |
|  |  |  |  |  |  |  | 189.0 | 30 | 6 |
|  |  |  |  |  |  |  | 203.0 | 30 | 6 |
| Deoxynivalenol ^13^C15 | 4.70 | [M+H]^+^ | 312.2 | 60 | 3 | 20 | 144.7 | 30 | 6 |
|  |  |  |  |  |  |  | **132.0** | **30** | **6** |
|  |  |  |  |  |  |  | 170.9 | 30 | 6 |
| Dimethoate | 4.77 | [M+H]^+^ | 230.0 | 30 | 3 | 20 | **124.9** | **30** | **6** |
|  |  |  |  |  |  |  | 142.9 | 30 | 6 |
|  |  |  |  |  |  |  | 199.0 | 30 | 6 |
| Clothianidin-desmethyl | 4.81 | [M+H]^+^ | 236.8 | 45 | 10 | 15 | **72.9** | **40** | **9** |
|  |  |  |  |  |  |  | 132.0 | 40 | 9 |
|  |  |  |  |  |  |  | 154.0 | 40 | 9 |
| 3-acetyldeoxynivalenol | 4.88 | [M+H]^+^ | 339.3 | 60 | 3 | 20 | **203.3** | **30** | **6** |
|  |  |  |  |  |  |  | 212.9 | 30 | 6 |
|  |  |  |  |  |  |  | 231.1 | 30 | 6 |
| Clothianidin | 4.91 | [M+H]^+^ | 250.1 | 30 | 3 | 10 | **169.0** | **15** | **9** |
|  |  |  |  |  |  |  | 131.9 | 30 | 9 |
| Imidacloprid | 4.95 | [M+H]^+^ | 256.0 | 45 | 3 | 10 | 175.2 | 45 | 3 |
|  |  |  |  |  |  |  | **209.1** | **30** | **9** |
| 15-acetyldeoxynivalenol | 4.95 | [M+H]^+^ | 339.0 | 30 | 3 | 20 | 187.2 | 30 | 6 |
|  |  |  |  |  |  |  | **137.0** | **30** | **6** |
| Acetamiprid-desmethyl | 5.05 | [M+H]^+^ | 209.1 | 60 | 3 | 15 | **126.0** | **45** | **9** |
|  |  |  |  |  |  |  | 104.7 | 45 | 6 |
| Imidacloprid-Olefin | 5.10 | [M+H]^+^ | 267.1 | 95 | 21 | 10 | 171.0 | 30 | 6 |
|  |  |  |  |  |  |  | 205.1 | 45 | 6 |
|  |  |  |  |  |  |  | **236.1** | **30** | **3** |
| Acetamiprid | 5.28 | [M+H]^+^ | 223.2 | 45 | 6 | 10 | **126.1** | **30** | **9** |
|  |  |  |  |  |  |  | 90.0 | 45 | 6 |
|  |  |  |  |  |  |  | 99.0 | 45 | 6 |
| Aflatoxin G2 | 5.75 | [M+H]^+^ | 331.3 | 75 | 3 | 20 | **245.1** | **30** | **6** |
|  |  |  |  |  |  |  | 257.3 | 30 | 6 |
|  |  |  |  |  |  |  | 228.8 | 30 | 6 |
| Aflatoxin M1 | 5.99 | [M+H]^+^ | 329.0 | 60 | 3 | 20 | 259.0 | 30 | 6 |
|  |  |  |  |  |  |  | **272.8** | **30** | **6** |
|  |  |  |  |  |  |  | 229.2 | 30 | 6 |
| Aflatoxin G1 | 6.16 | [M+H]^+^ | 329.0 | 75 | 3 | 20 | **243.2** | **30** | **6** |
|  |  |  |  |  |  |  | 215.1 | 30 | 6 |
|  |  |  |  |  |  |  | 255.0 | 30 | 6 |
| Ochratoxin alpha | 6.16 | [M-H]^-^ | 255.0 | -45 | -6 | -30 | 166.6 | -45 | -9 |
|  |  |  |  |  |  |  | **210.5** | **-30** | **-9** |
| Aflatoxin B2 | 6.55 | [M+H]^+^ | 315.2 | 75 | 3 | 20 | **259.0** | **30** | **6** |
|  |  |  |  |  |  |  | 227.2 | 30 | 6 |
|  |  |  |  |  |  |  | 243.1 | 45 | 6 |
| Aflatoxin B1 | 6.90 | [M+H]^+^ | 313.0 | 75 | 3 | 20 | **241.1** | **45** | **6** |
|  |  |  |  |  |  |  | 229.1 | 30 | 6 |
|  |  |  |  |  |  |  | 296.6 | 30 | 6 |
| TCPY | 7.50 | [M+H]^+^ | 198.1 | 60 | 6 | 10 | **106.9** | **45** | **9** |
|  |  |  |  |  |  |  | 133.9 | 30 | 6 |
| HT2 toxin | 7.66 | [M+Na]^+^ | 447.3 | 60 | 3 | 20 | 285.2 | 45 | 9 |
|  |  |  |  |  |  |  | **345.2** | **30** | **6** |
| Azoxystrobin acid | 7.95 | [M-H]^-^ | 388.0 | -30 | -3 | -20 | **211.7** | **-15** | **-9** |
|  |  |  |  |  |  |  | 168.0 | -45 | -9 |
| T2 toxin | 8.26 | [M+Na]^+^ | 489.3 | 75 | 3 | 20 | 326.9 | 30 | 6 |
|  |  |  |  |  |  |  | 267.2 | 30 | 6 |
|  |  |  |  |  |  |  | **387.2** | **30** | **6** |
| Ochratoxin B | 8.58 | [M+H]^+^ | 370.1 | 60 | 3 | 20 | **205.0** | **45** | **9** |
|  |  |  |  |  |  |  | 222.8 | 30 | 9 |
| β-Zearalenol | 8.76 | [M-H]^-^ | 318.9 | -75 | -3 | -20 | **174.0** | **-30** | **-6** |
|  |  |  |  |  |  |  | 159.6 | -30 | -6 |
|  |  |  |  |  |  |  | 187.0 | -30 | -6 |
| Azoxystrobin | 8.80 | [M+H]^+^ | 404.2 | 50 | 5 | 10 | 344.1 | 35 | 8 |
|  |  |  |  |  |  |  | **372.1** | **20** | **8** |
|  |  |  |  |  |  |  | 329.1 | 35 | 8 |
| Permethrinic acid | 8.88 | [M-H]^-^ | 207.1 | -30 | -3 | -20 | **206.4** | **-15** | **9** |
|  |  |  |  |  |  |  | 170.5 | -15 | 9 |
| Tebuconazole-hydroxy | 8.95 | [M+H]^+^ | 324.0 | 75 | 3 | 20 | **179.2** | **30** | **9** |
|  |  |  |  |  |  |  | 165.3 | 30 | 6 |
|  |  |  |  |  |  |  | 151.1 | 30 | 6 |
| Zearalanone | 9.05 | [M-H]^-^ | 319.2 | -75 | -3 | -20 | **204.7** | **-45** | **-9** |
|  |  |  |  |  |  |  | 186.6 | -30 | -9 |
| 3-Phenoxybenzoic acid | 9.14 | [M-H]^-^ | 213.2 | -75 | -3 | -20 | **93.0** | **-30** | **-3** |
| Ochratoxin A | 9.31 | [M+H]^+^ | 403.9 | 60 | 3 | 20 | **238.8** | **30** | **6** |
|  |  |  |  |  |  |  | 221.2 | 45 | 6 |
| Zearalenone | 9.40 | [M-H]^-^ | 317.0 | -75 | -3 | -20 | **174.6** | **-30** | **-6** |
|  |  |  |  |  |  |  | 186.6 | -30 | -6 |
|  |  |  |  |  |  |  | 148.7 | -30 | -6 |
| α-Zearalenol | 9.58 | [M-H]^-^ | 319.0 | -75 | -3 | -20 | **174.0** | **-30** | **-9** |
|  |  |  |  |  |  |  | 187.9 | -30 | -6 |
|  |  |  |  |  |  |  | 159.3 | -30 | -6 |
| Pirimiphos-Methyl | 9.65 | [M+H]^+^ | 306.3 | 45 | 12 | 20 | 164.1 | 45 | 9 |
|  |  |  |  |  |  |  | **136.1** | **30** | **6** |
| Tebuconazole | 10.10 | [M+H]^+^ | 308.1 | 60 | 6 | 20 | **125.1** | **45** | **9** |
|  |  |  |  |  |  |  | 151.4 | 30 | 6 |
|  |  |  |  |  |  |  | 139.0 | 30 | 6 |
| Chlorpyrifos methyl | 10.77 | [M+H]^+^ | 324.0 | 60 | 3 | 10 | 142.8 | 45 | 9 |
|  |  |  |  |  |  |  | **214.0** | **45** | **6** |
| Enniatin B | 11.24 | [M+H]^+^ | 640.4 | 81 | 9 | 18 | **196.2** | **35** | **4** |
|  |  |  |  |  |  |  | 214.2 | 37 | 4 |
| Enniatin B1 | 11.30 | [M+H]^+^ | 654.4 | 81 | 7 | 30 | **196.4** | **39** | **6** |
|  |  |  |  |  |  |  | 210.1 | 33 | 6 |
| Chlorpirifos | 11.35 | [M+H]^+^ | 349.9 | 60 | 3 | 20 | 152.8 | 30 | 6 |
|  |  |  |  |  |  |  | **198.0** | **30** | **6** |
|  |  |  |  |  |  |  | 213.9 | 30 | 6 |
| Beauvericin | 11.35 | [M+H]^+^ | 784.5 | 81 | 9 | 26 | **244.2** | **39** | **4** |
|  |  |  |  |  |  |  | 262.2 | 37 | 4 |
| Enniatin A1 | 11.53 | [M+H]^+^ | 668.4 | 81 | 11 | 18 | **210.2** | **35** | **6** |
|  |  |  |  |  |  |  | 228.2 | 35 | 4 |
| Cypermethrin | 11.87 | [M+H]^+^ | 416.0 | 45 | 3 | 10 | **191.1** | **15** | **6** |
|  |  |  |  |  |  |  | 163.0 | 30 | 6 |
| Enniatin A | 11.90 | [M+H]^+^ | 682.4 | 75 | 9 | 25 | **210.2** | **35** | **4** |
|  |  |  |  |  |  |  | 228.2 | 35 | 4 |

*Ions highlighted in bold correspond to the quantification transition; Ions highlighted in red correspond to transition interferences

Abbreviations: CE: Collision energy; CEP: Cell entrance potential; CXP: Collision cell exit potential; DEAMPY: 2-diethylamino-6-methyl-4-pyrimidinol; DEDTP: diethyl dithiophosphate; DEP: diethyl phosphate; DETP: diethyl thiophosphate; DMDTP: dimethyl dithiophosphate; DMP: dimethyl phosphate; DMTP: dimethyl thiophosphate; DP: Declustering potential; EP: Entrance potential; TCPY: 3,5,6-Trichloro-2-pyridinol

**Figure captions**

**Fig S1.1** Extracted ion chromatogram of studied compounds (MRM in positive mode) at 100 µg/L using a Zorbax Eclipse Plus C18 column (100 x 2.1 mm, 1.8 µm particle size)

**Fig S1.2** Extracted ion chromatogram of studied compounds (MRM in negative mode) at 100 µg/L using a Zorbax Eclipse Plus C18 column (100 x 2.1 mm, 1.8 µm particle size)

**Fig S2.1** Extracted ion chromatogram of studied compounds (MRM in positive mode) at 100 µg/L using a Acquity HSS T3 C18 column (150 x 2.1 mm, 1.8 µm particle size)

**Fig S2.2** Extracted ion chromatogram of studied compounds (MRM in negative mode) at 100 µg/L using a Acquity HSS T3 C18 column (150 x 2.1 mm, 1.8 µm particle size)

**Fig S3.1** Extracted ion chromatogram of studied compounds (MRM in positive mode) at 100 µg/L using a Cosmocore 2.6PBr column (100 x 2.1 mm, 2.6 µm article size)

**Fig S3.2** Extracted ion chromatogram of studied compounds (MRM in negative mode) at 100 µg/L using a Cosmocore 2.6PBr column (100 x 2.1 mm, 2.6 µm article size)

**Fig S4** PCA for pesticides regarding high consumption *vs* low consuming of fruits and vegetables

**Fig S5** PCA for pesticides regarding farmers *vs* general population

**Fig S6** PCA for NEOs and PYs regarding farmers *vs* general population

**Fig S7** PCA for mycotoxins regarding farmers *vs* general population

**Fig S8** PCA for mycotoxins regarding high consumption *vs* low consuming of cereals and nuts

**Fig S9** VIP for mycotoxins regarding high consumption *vs* low consuming of cereals and nuts


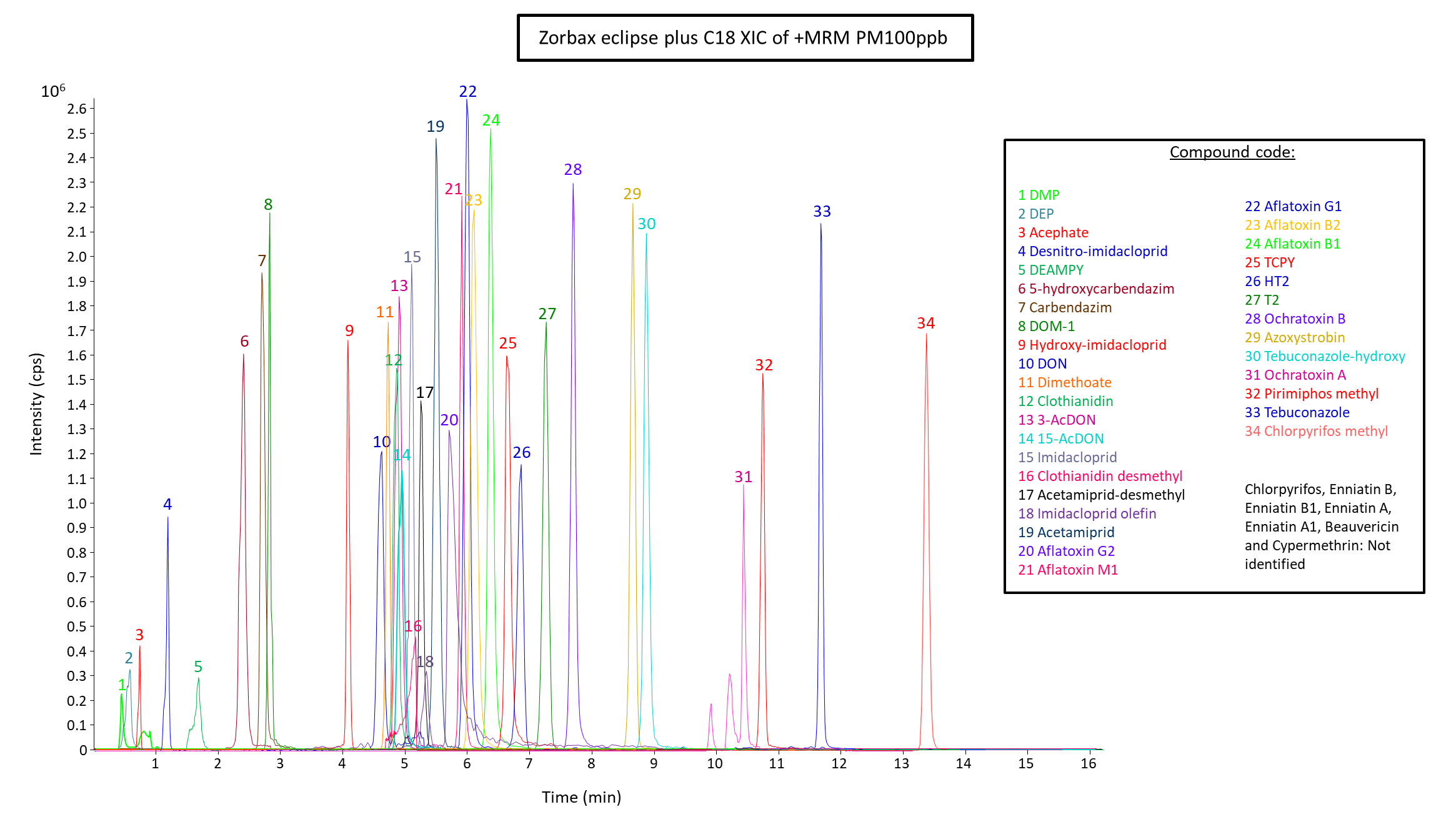


**Fig S1.1** Extracted ion chromatogram of studied compounds (MRM in positive mode) at 100 µg/L using a Zorbax Eclipse Plus C18 column (100 x 2.1 mm, 1.8 µm particle size)


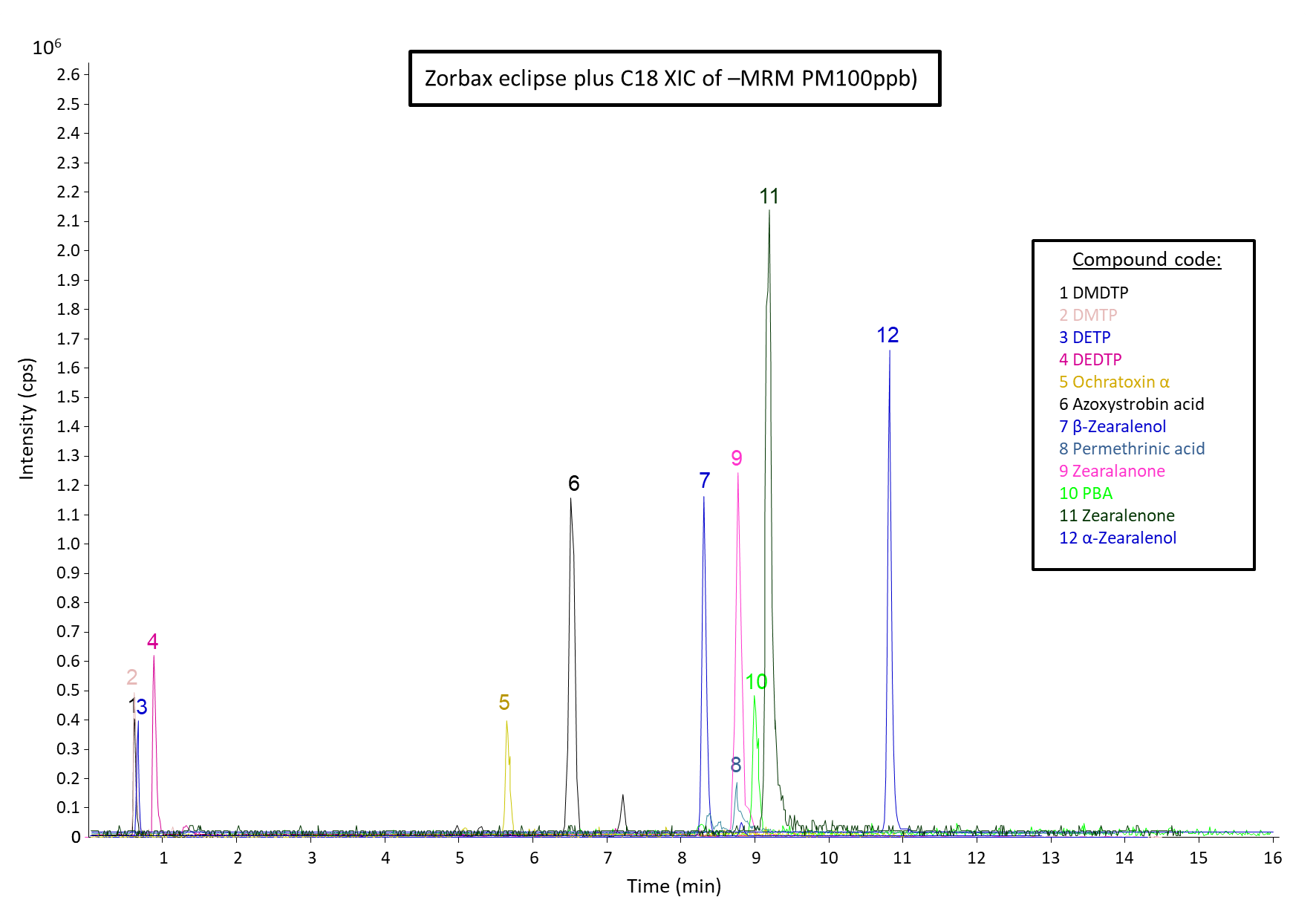


**Fig S1.2** Extracted ion chromatogram of studied compounds (MRM in negative mode) at 100 µg/L using a Zorbax Eclipse Plus C18 column (100 x 2.1 mm, 1.8 µm particle size)

**
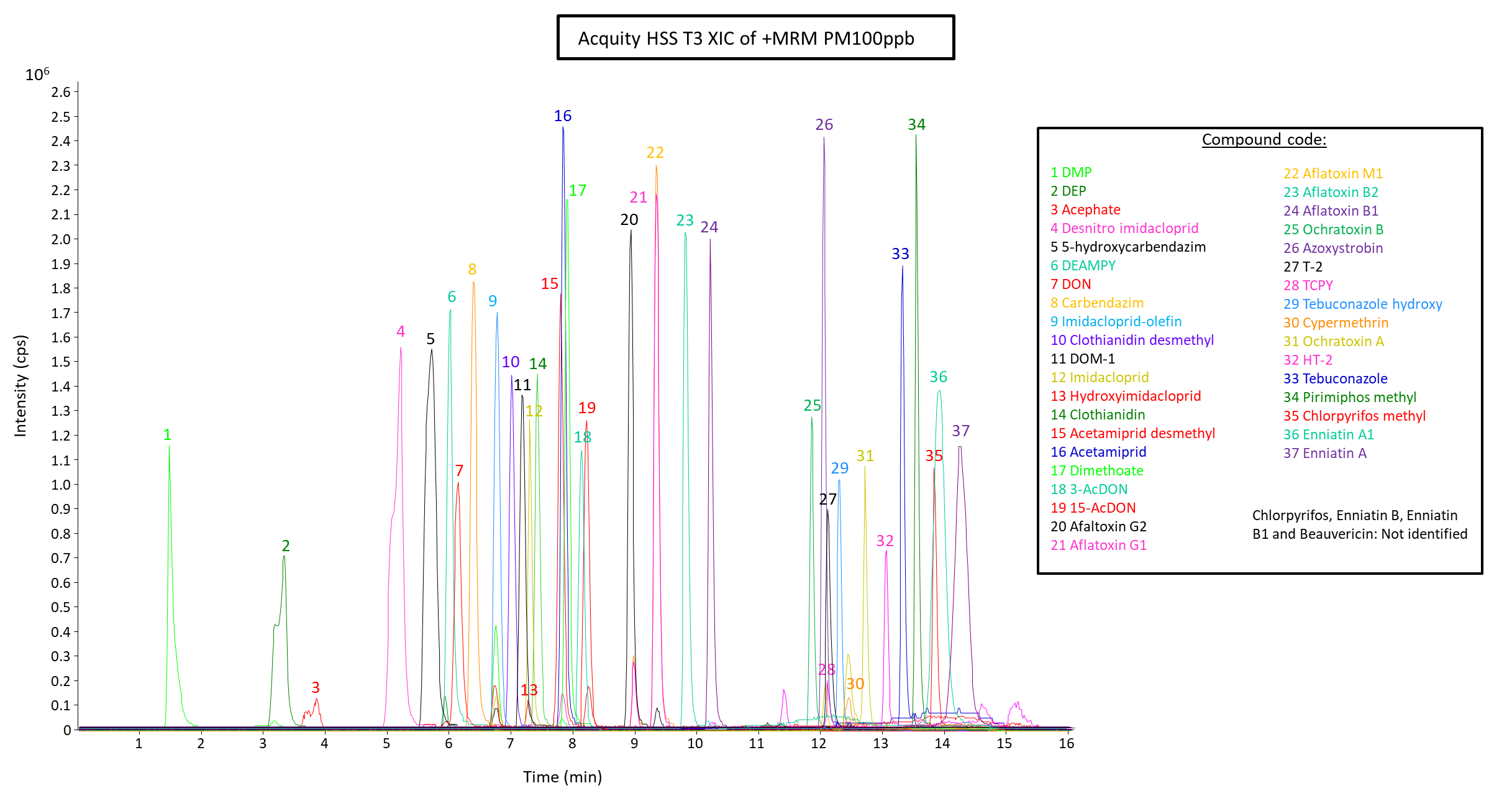
**

**Fig S2.1** Extracted ion chromatogram of studied compounds (MRM in positive mode) at 100 µg/L using a Acquity HSS T3 C18 column (150 x 2.1 mm, 1.8 µm particle size)


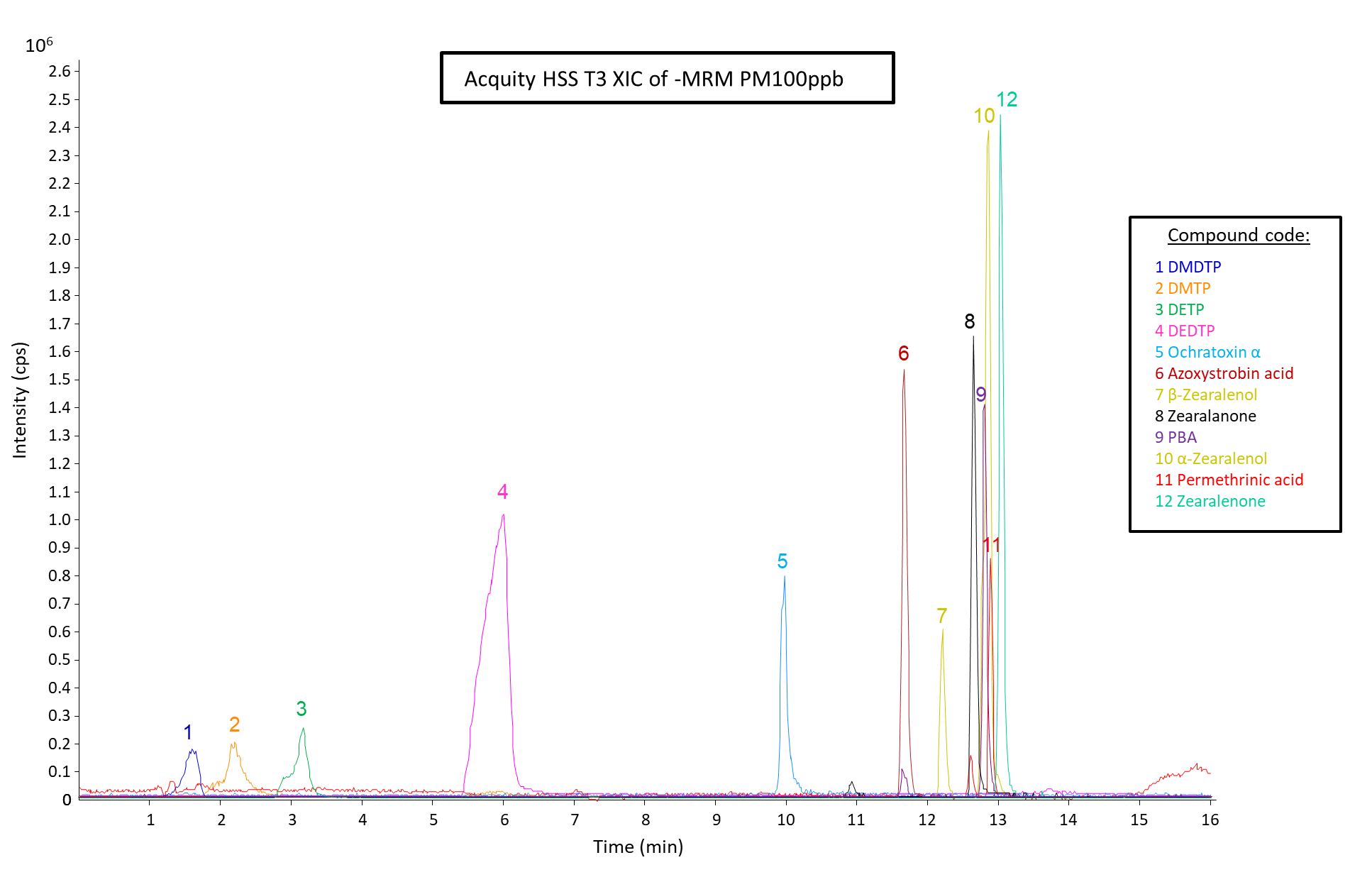


**Fig S2.2** Extracted ion chromatogram of studied compounds (MRM in negative mode) at 100 µg/L using a Acquity HSS T3 C18 column (150 x 2.1 mm, 1.8 µm particle size)


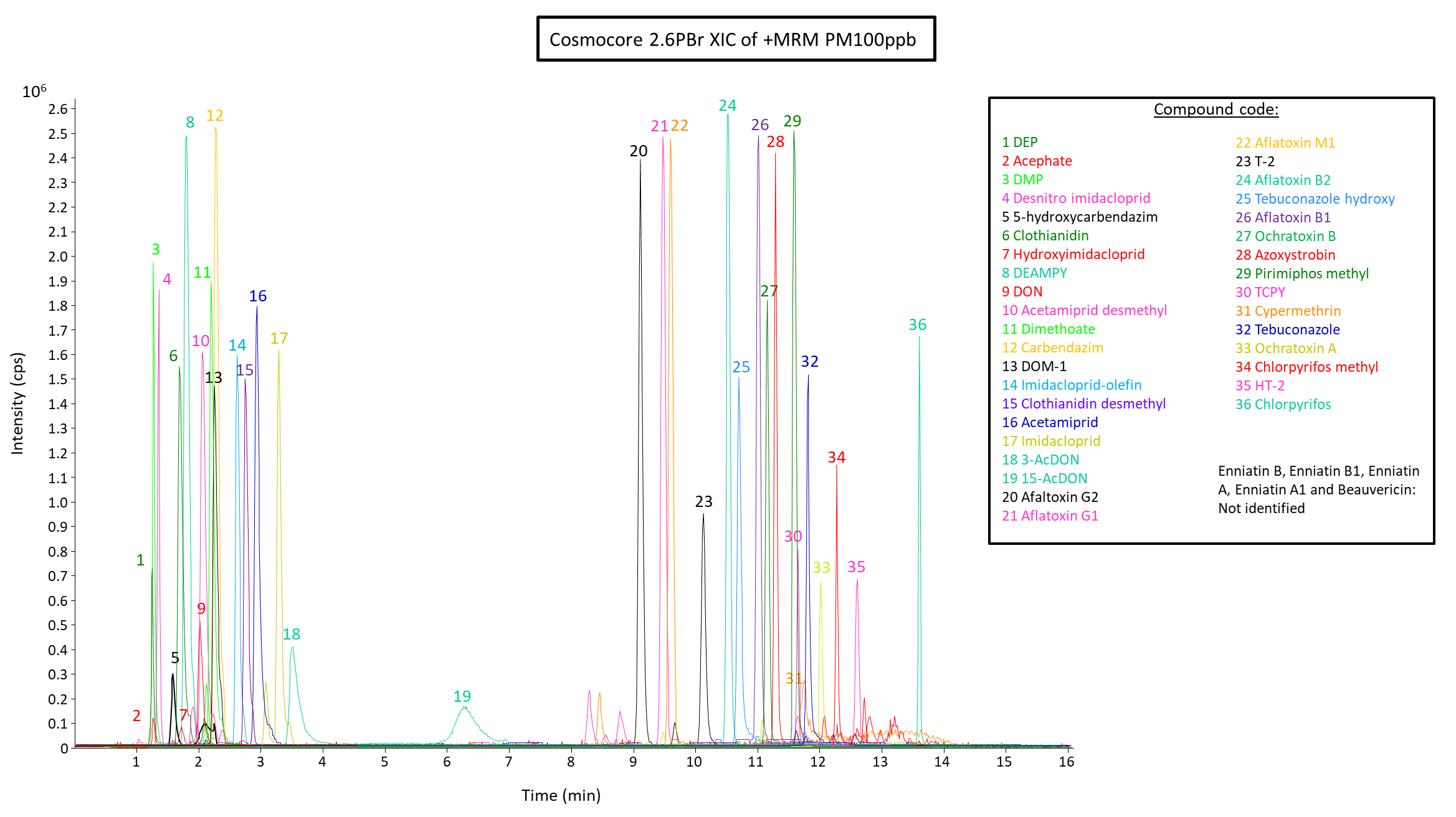


**Figure S3.1.** Extracted ion chromatogram of studied compounds (MRM in positive mode) at 100 µg/L using a Cosmocore 2.6PBr column (100 x 2.1 mm, 2.6 µm article size)


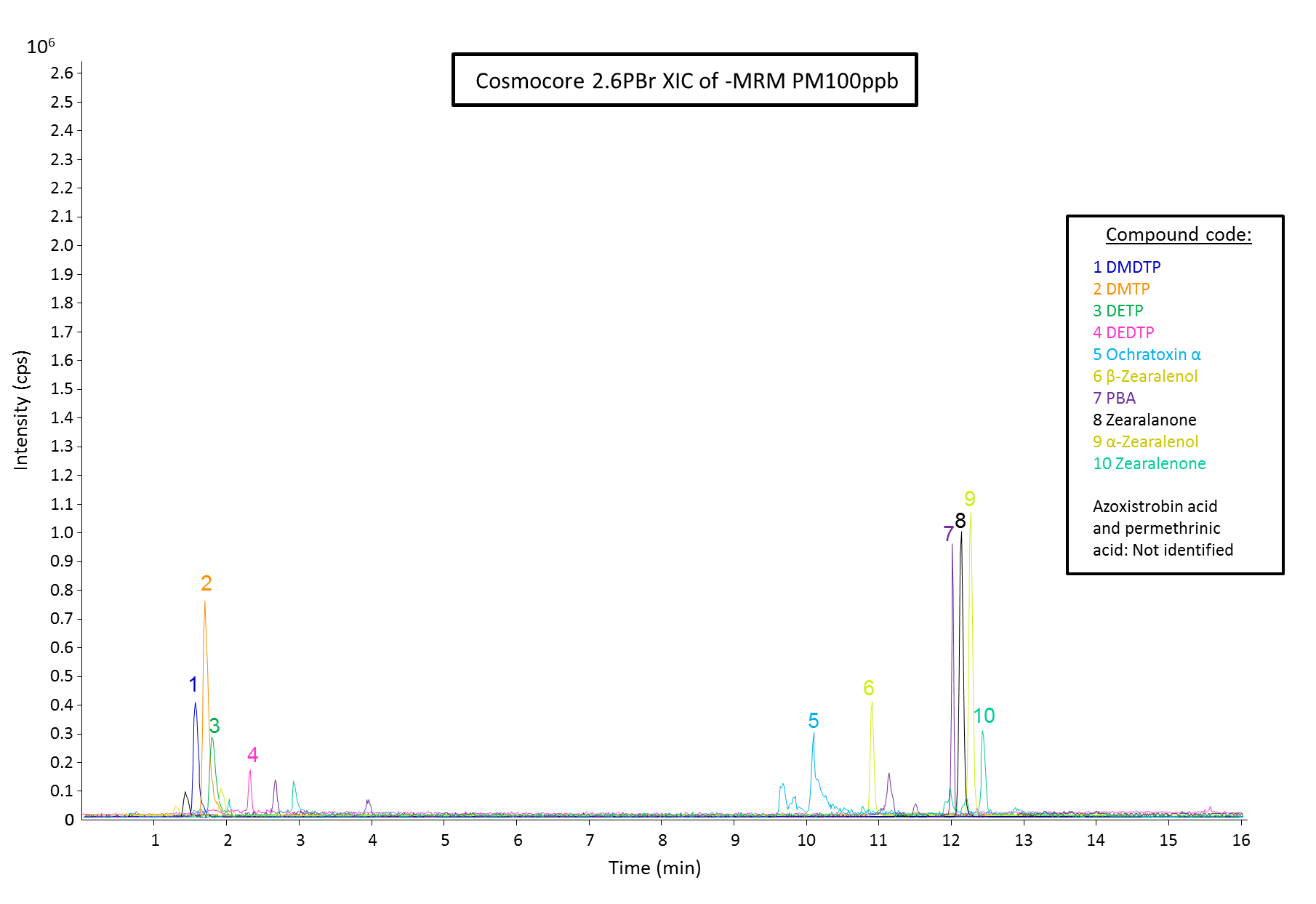


**Fig S3.2** Extracted ion chromatogram of studied compounds (MRM in negative mode) at 100 µg/L using a Cosmocore 2.6PBr column (100 x 2.1 mm, 2.6 µm article size)


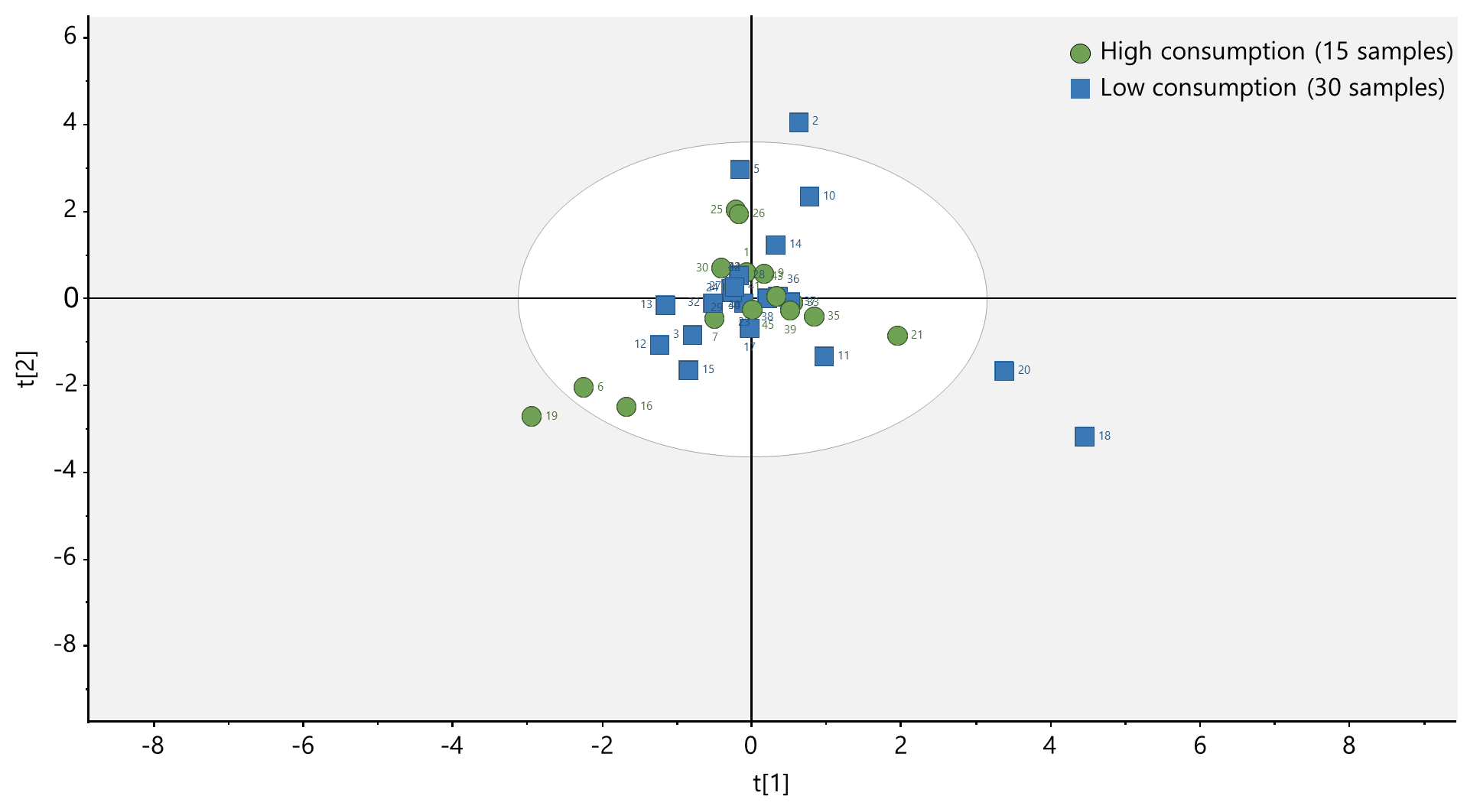


**Fig S4** PCA for pesticides regarding high *vs* low consumption of fruits and vegetables


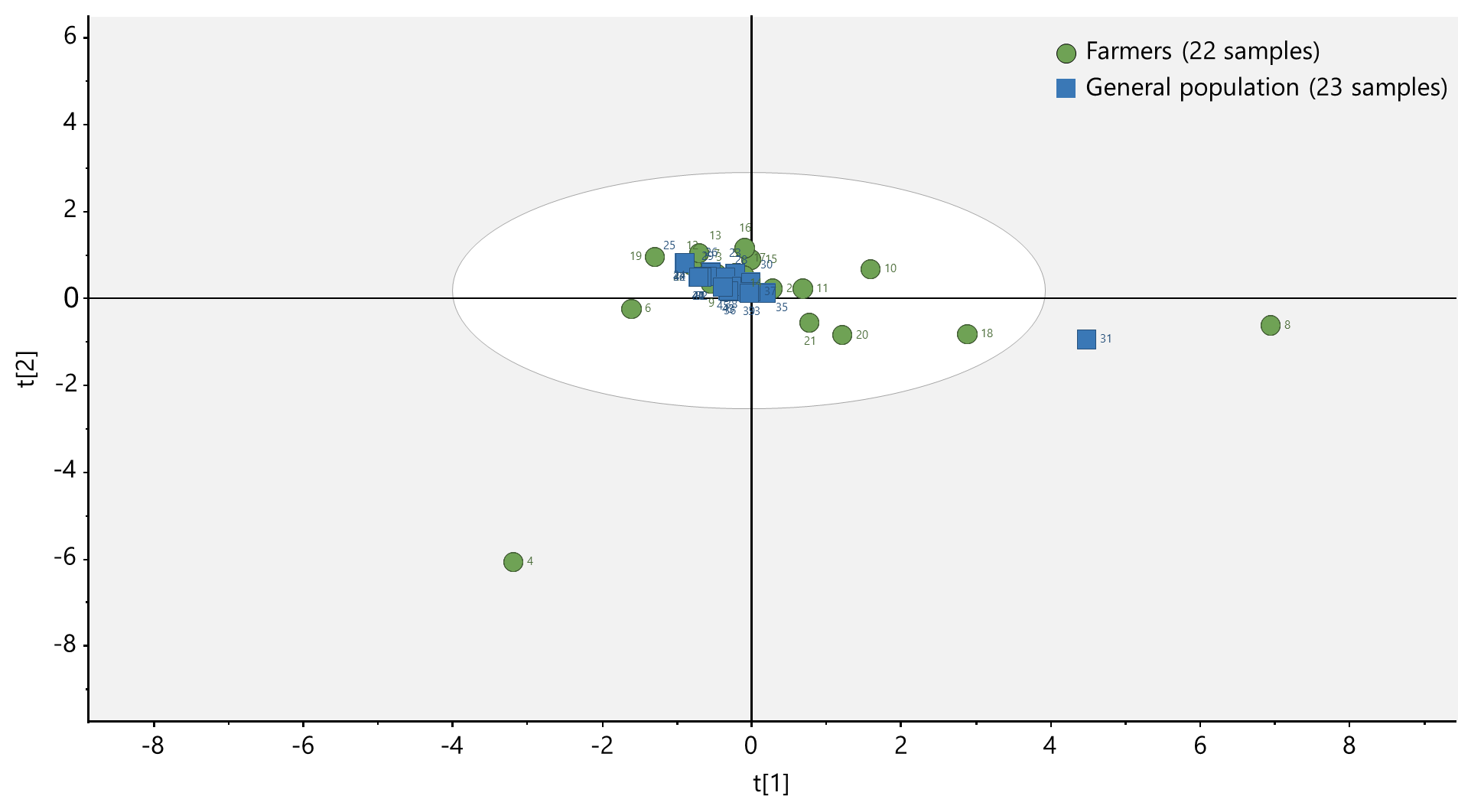


**Fig S5** PCA for pesticides regarding farmers *vs* general population


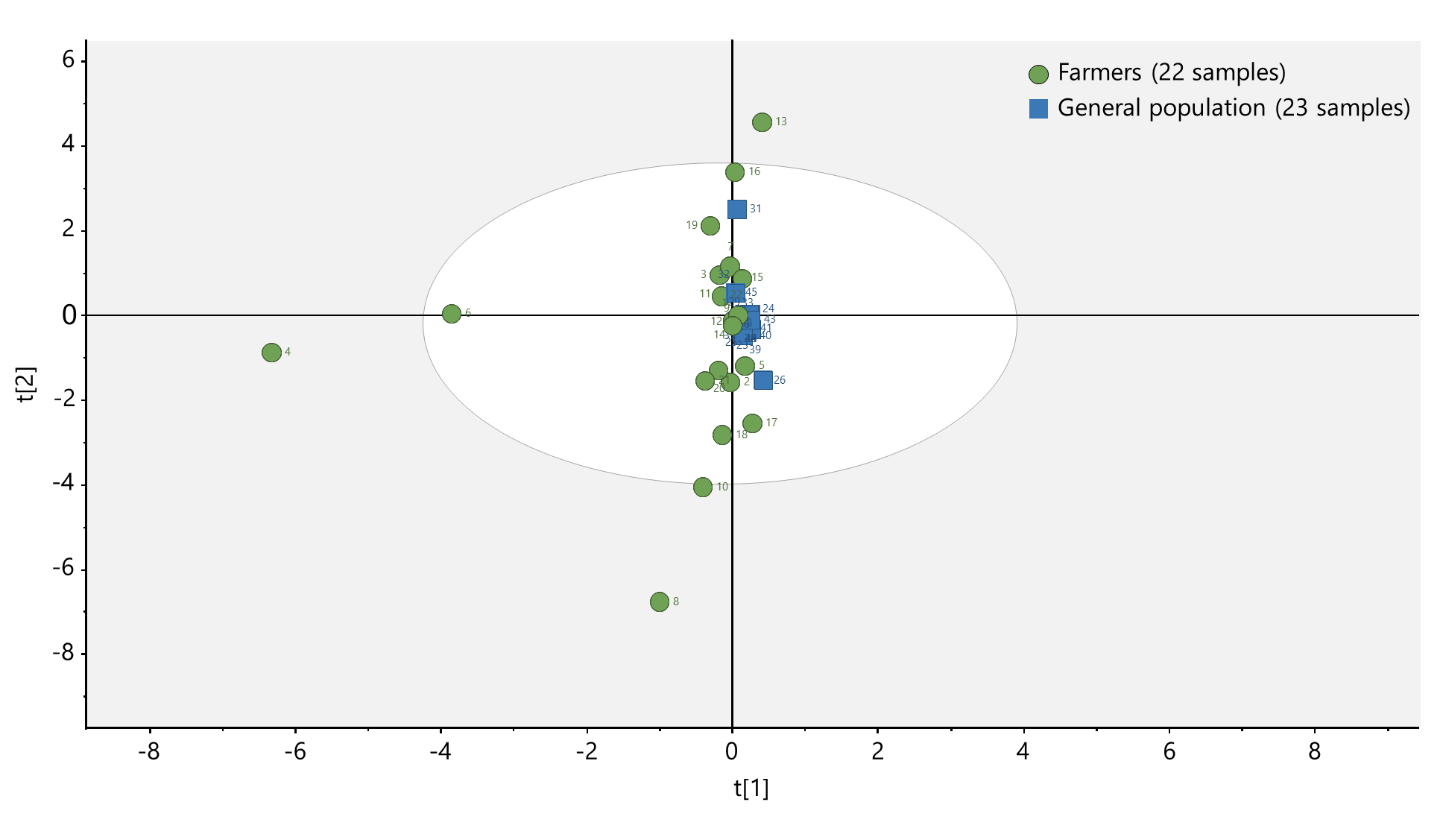


**Fig S6** PCA for NEOs and PYs regarding farmers *vs* general population


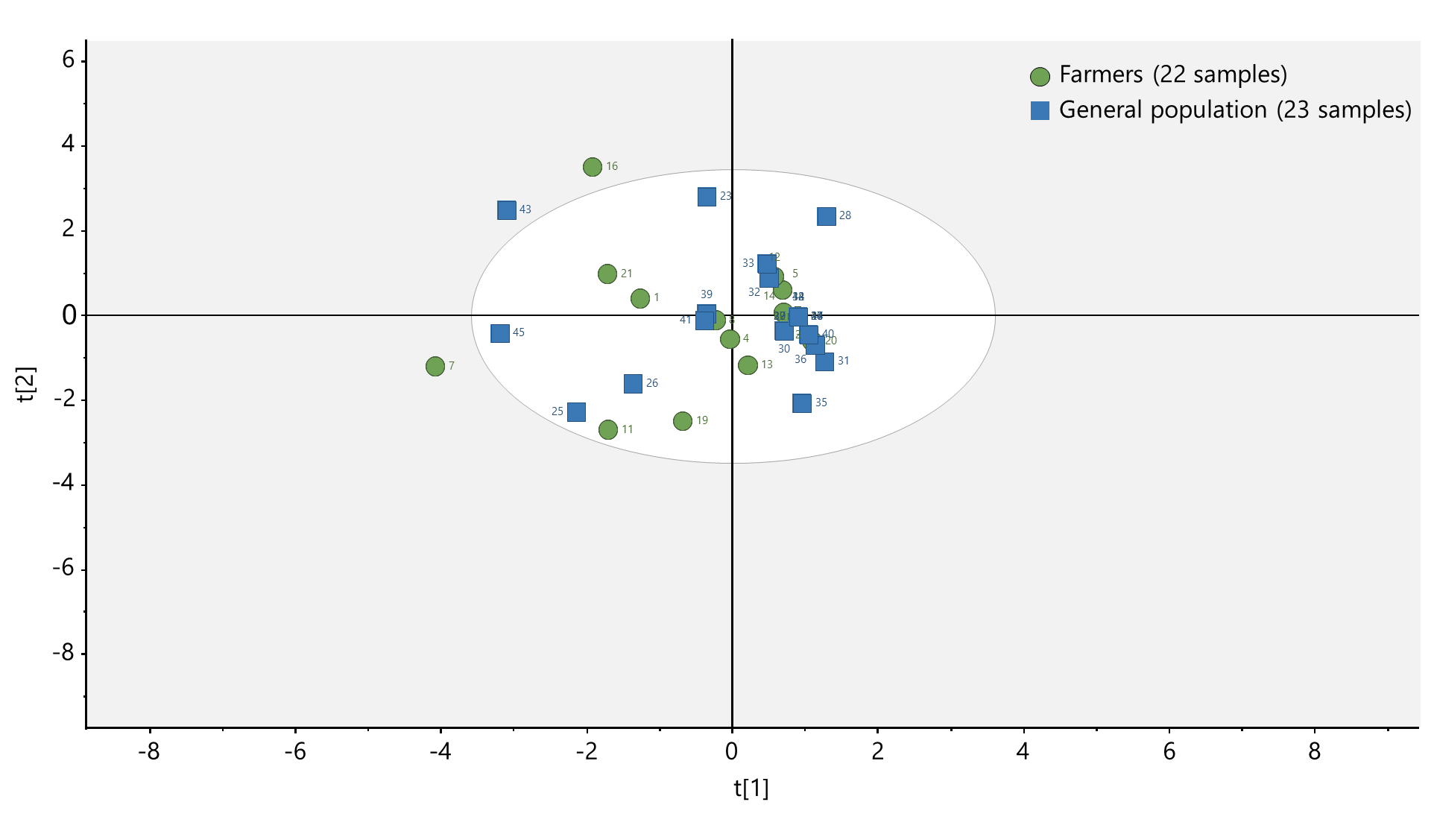


**Fig S7** PCA for mycotoxins regarding farmers *vs* general population


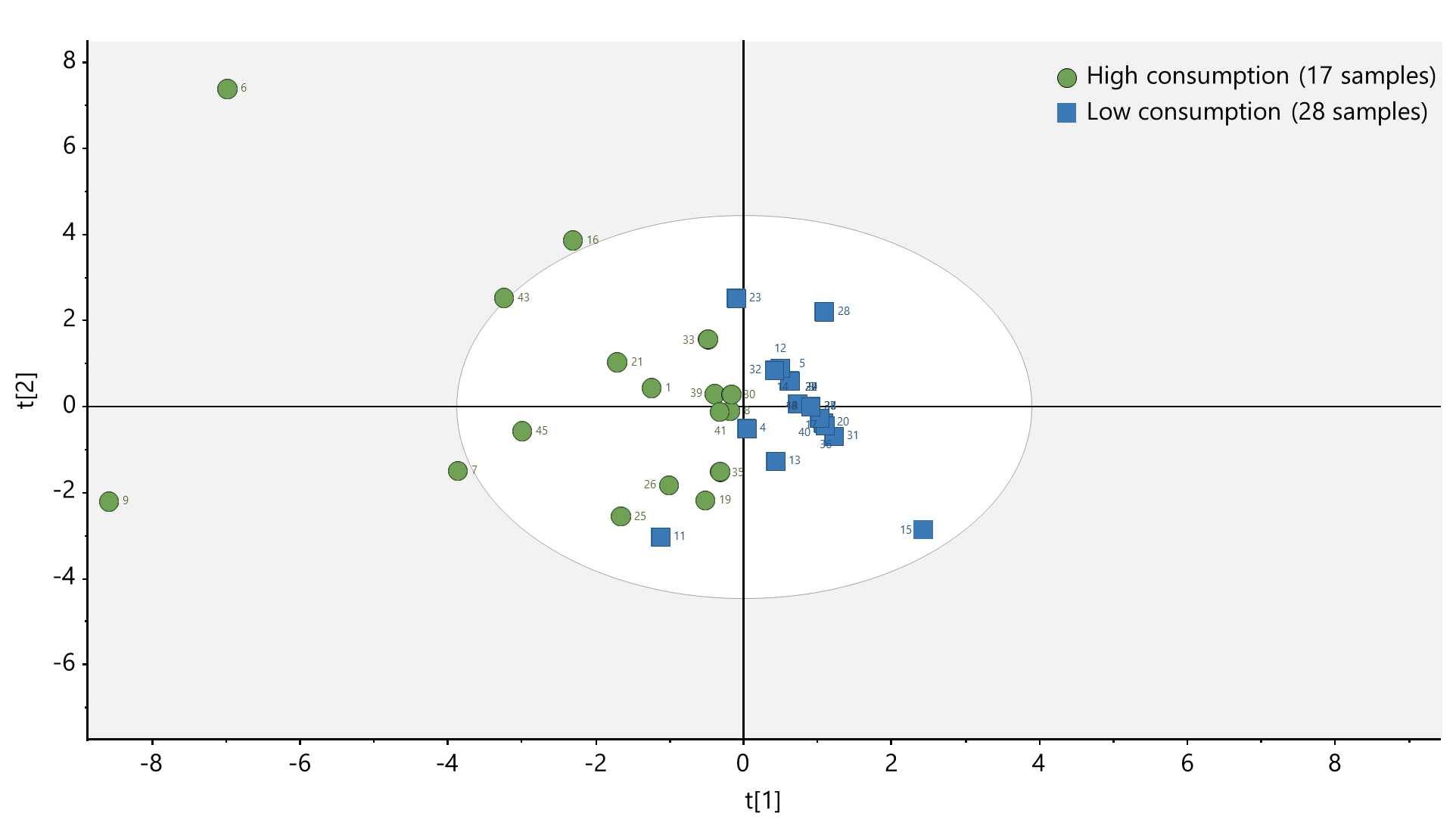


**Fig S8** PCA for mycotoxins regarding high *vs* low consumption of cereals and nuts


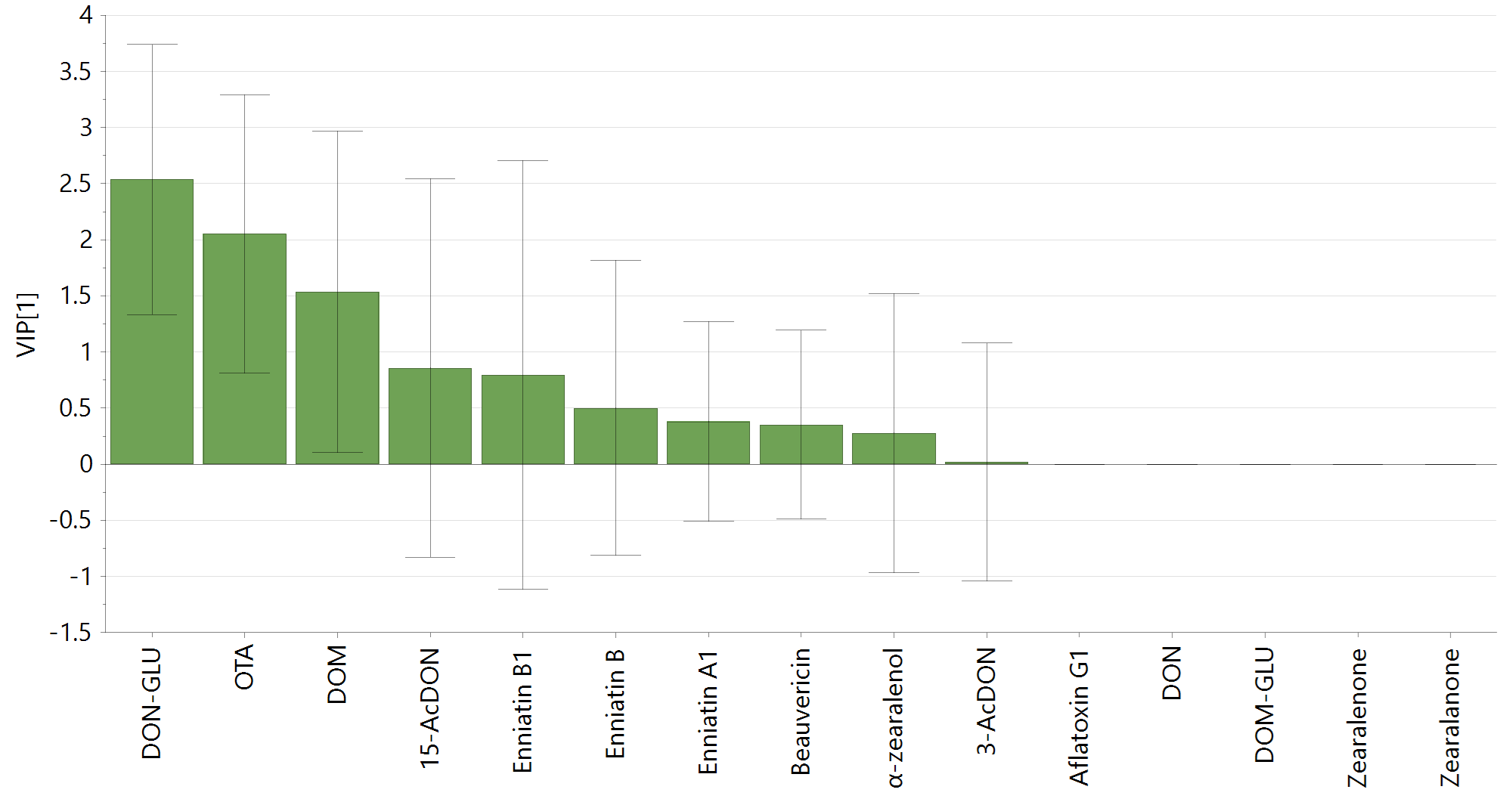


**Fig S9** VIP for mycotoxins regarding high *vs* low consumption of cereals and nuts
